# The neighbourhood built environment and health-related fitness: a narrative systematic review

**DOI:** 10.1186/s12966-022-01359-0

**Published:** 2022-09-24

**Authors:** Levi Frehlich, Chelsea D. Christie, Paul E. Ronksley, Tanvir C. Turin, Patricia Doyle-Baker, Gavin R. McCormack

**Affiliations:** 1 Department of Community Health Sciences, Cumming School of Medicine, University of Calgary, Calgary, Canada; 2 Department of Family Medicine, Cumming School of Medicine, University of Calgary, Calgary, Canada; 3 Faculty of Kinesiology, University of Calgary, Calgary, Canada; 4 School of Architecture, Planning and Landscape, University of Calgary, Calgary, Canada; 5 Faculty of Sport Sciences, Waseda University, Shinjuku City, Japan

**Keywords:** Built environment, Neighbourhood, Physical activity, Health-related fitness

## Abstract

**Background:**

There is increasing evidence demonstrating the importance of the neighbourhood built environment in supporting physical activity. Physical activity provides numerous health benefits including improvements in health-related fitness (i.e., muscular, cardiorespiratory, motor, and morphological fitness). Emerging evidence also suggests that the neighbourhood built environment is associated with health-related fitness. Our aim was to summarize evidence on the associations between the neighbourhood built environment and components of health-related fitness in adults.

**Methods:**

We undertook a systematic review following PRISMA guidelines. Our data sources included electronic searches in MEDLINE, Embase, CINAHL, Web of Science, SPORTDiscus, Environment Complete, ProQuest Dissertations and Theses, and Transport Research International Documentation from inception to March 2021. Our eligibility criteria consisted of observational and experimental studies estimating associations between the neighbourhood built environment and health-related fitness among healthy adults (age ≥ 18 years). Eligible studies included objective or self-reported measures of the neighbourhood built environment and included either objective or self-reported measures of health-related fitness. Data extraction included study design, sample characteristics, measured neighbourhood built environment characteristics, and measured components of health-related fitness. We used individual Joanna Briggs Institute study checklists based on identified study designs. Our primary outcome measure was components of health-related fitness (muscular; cardiorespiratory; motor, and morphological fitness).

**Results:**

Twenty-seven studies (sample sizes = 28 to 419,562; 2002 to 2020) met the eligibility criteria. Neighbourhood destinations were the most consistent built environment correlate across all components of health-related fitness. The greatest number of significant associations was found between the neighbourhood built environment and morphological fitness while the lowest number of associations was found for motor fitness. The neighbourhood built environment was consistently associated with health-related fitness in studies that adjusted for physical activity.

**Conclusion:**

The neighbourhood built environment is associated with health-related fitness in adults and these associations may be independent of physical activity. Longitudinal studies that adjust for physical activity (including resistance training) and sedentary behaviour, and residential self-selection are needed to obtain rigorous causal evidence for the link between the neighbourhood built environment and health-related fitness.

**Trial registration:**

Protocol registration: PROSPERO number CRD42020179807.

**Supplementary Information:**

The online version contains supplementary material available at 10.1186/s12966-022-01359-0.

## Background

Participation in regular physical activity is associated with a reduced risk of developing diabetes [1], cardiovascular disease [2], certain cancers [3] and premature mortality [4]. Notably, physical activity is also positively associated with health-related fitness [5]. Health-related fitness reflects physiological attributes that delay the onset of morbidity from diseases that may result from living a physically inactive lifestyle [6]. Traditional definitions of health-related fitness (i.e., cardiorespiratory endurance, muscular endurance, muscular strength, body composition and flexibility) [7] have since been updated to be more encompassing [8]. Current definitions of health-related fitness are multidimensional and include morphologic (e.g., body composition or flexibility) muscular (e.g., grip strength or endurance), cardiorespiratory (e.g., $\dot{V}{O}_2\ \mathit{\max}$ or sustained cardiorespiratory capacity), motor (e.g., balance or proprioceptive activity), and metabolic (e.g., blood lipid or glucose levels) components [6]. After controlling for body mass index (BMI) and waist circumference, objective measures of body composition (including the distribution of adipose tissue) have been linked to incident cardiovascular disease [9]. Findings from a meta-analysis demonstrated that decreases in grip strength were associated with an increased risk of all-cause and cardiovascular mortality [10]. Associations between lower grip strength in mid-life with functional limitations and disability in older adulthood have also been observed [11]. Cardiorespiratory fitness, has been shown to be associated with cardiovascular disease risk in adults [12].

Higher intensity physical activity can improve muscular [13], cardiorespiratory [13], and morphological fitness [14]; however, even lower intensity activities, such as walking, may improve health-related fitness [15]. Qualitative [16] and quantitative [17] evidence consistently demonstrates links between neighbourhood built environment and physical activity. Key built environment features that support physical activity include density (i.e., residential or population), connectivity (i.e., many potential routes, short block sizes, many intersections), and land uses (i.e., recreational and utilitarian destinations) [16, 17]. Giles-Corti et al. developed [18] and later expanded [19] a framework positing potential pathways by which the local built environment is associated with physical activity and health. The framework highlights important built characteristics including *design* (e.g., street layout and connectivity), *density* (e.g., compactness of residential population), *transit* (e.g., proximity and access), *destination proximity* (e.g., distance to local destinations), *diversity* (e.g., mixed residential, commercial, and recreational destinations), *desirability* (e.g., safety and aesthetics) and *distributed* features (e.g., resources equitably distributed across different populations) [18, 19]. Given the connections between the built environment and physical activity, and physical activity and health-related fitness, neighbourhood built environments may play a vital role in supporting health-related fitness in adults.

Health-related fitness can be influenced by genetic factors, lifestyle behaviours, personal attributes, and physical and social environments [8]. Notably, some evidence suggests that associations between the built environment and health-related fitness remain after controlling for physical activity [20–23]. The persistent relationship may reflect the presence of independent pathways between the built environment and health-related fitness, the existence of other mediators (e.g., sedentary behavior and diet), or inadequate adjustment for physical activity. For example, studies have found the availability of food destinations to be associated with morphological fitness [24] and sedentary time to be association with functional-related fitness in older adults [25]. Both sedentary behaviour and diet are associated with built environment [26–28].

While several studies have found significant associations between some features of the neighbourhood built environment and health-related fitness [23, 29–31], this literature has not been systematically synthesized nor critically evaluated. Therefore, the aim of this study is two-fold: (1) to summarize and critically appraise the existing literature on the associations between the neighbourhood built environment and health-related fitness in the adult general population, and; (2) to identify and summarize studies estimating the associations between the neighbourhood built environment and health-related fitness that also control for physical activity.

## Methods

This systematic review is based on a published study protocol [32], was registered in the International prospective register of systematic reviews (PROSPERO; ID number: CRD42020179807), and follows the Preferred Reporting Items for Systematic Reviews and Meta-Analyses (PRISMA) guidelines (Supplementary material; S1) [33]. We deviated from the protocol by having only one reviewer (LF) screen all initial titles and abstracts, however, two reviewers (LF and CC) screened the potentially relevant full-texts and collaboratively extracted study data (i.e., through a consensus approach).

### Search strategy

Databases were searched from inception to March 2021 with no language or location restrictions. MEDLINE (Ovid), Embase (Ovid), CINAHL (EBSCO), Web of Science, SPORTDiscus (EBSCO), and Environment Complete (EBSCO) were search for published evidence (Supplementary material; S2). Our search was supplemented with an exploration of unpublished evidence from ProQuest Dissertations and Theses. Finally, Transport Research International Documentation was also explored for relevant unpublished and published evidence.

### Study selection

Citations were collated and uploaded into Covidence (Covidence systematic review software, Veritas Health Innovation, Melbourne, Australia. Available at www.covidence.org) and duplicates were removed.

### Eligibility criteria

#### Types of studies

We included observational and experimental studies that reported on quantitative results. Our review excluded qualitative studies and literature reviews.

#### Participants

We included studies undertaken with healthy adults (≥18 years of age). We excluded studies undertaken with children or adolescents, athletes, or clinical populations.

#### Exposure(s)

Exposure variables eligible for inclusion were built environment characteristics measured using objective (e.g., Geographical Information Systems or environmental audits) or self-reported (e.g., questionnaire) approaches.

#### Outcomes

Eligible studies included objective (e.g., researcher-administered field tests or laboratory testing) or self-reported measures (e.g., survey questionnaires) of health-related fitness. Health-related fitness included any measures of muscular, cardiorespiratory, motor, and morphological fitness. We excluded metabolic fitness because compared to the other components of health-related fitness, recent systematic reviews have summarized the associations between the built environment and cardio-metabolic health [34–39]. Within morphological fitness, outcomes of body composition were included if studies distinguished between fat and fat-free mass (e.g., body fat percentage), but they were excluded if they could not (e.g., BMI and waist-to-hip-ratio).

### Data extraction

Data extraction included title, author, year of study, journal, study design, geographical location, sample size, mean age and age range, participant sex/gender distribution, data collection date, study duration, statistical technique, and estimate type(s), whether the built environment was objectively-measured or self-reported, whether the components of health-related fitness were objectively-measured or self-reported, the built environment characteristics measured, the component of health-related fitness measured, built environment exposure, covariates present in the adjusted results, whether adjustment was made for physical activity, and the main study findings.

### Assessment of study quality

Study quality was assessed using the Joanna Briggs Institute (JBI) critical appraisal tools for cross-sectional [40] (8 items), quasi-experimental [41] (9-items) or cohort [40] (12-items) studies. We used three specific study quality tools to accommodate the different studies designs that we expected to encounter in this literature [17, 42–44].

### Data synthesis

A narrative synthesis was completed by categorizing perceived or objectively measured individual (e.g., street connectivity) or index (e.g., walkability) built environment measures as well as perceived or objectively measured components of health-related fitness (e.g., cardiorespiratory fitness). Using an established framework [18, 19], built environment characteristics were grouped into one of seven feature categories (i.e., design, density, transit, destination proximity, diversity, desirability, and distributed). We also added an eighth category – “Composite or Other” features – which included measures that combined individual built environment features into a single index or score (e.g., “walkability”) or where a single built environment variable spanned multiple features (e.g., urban infrastructure improvement). Statistically significant positive, negative, and non-significant associations were summarized.

## Results

### Study identification

**Fig. 1:**
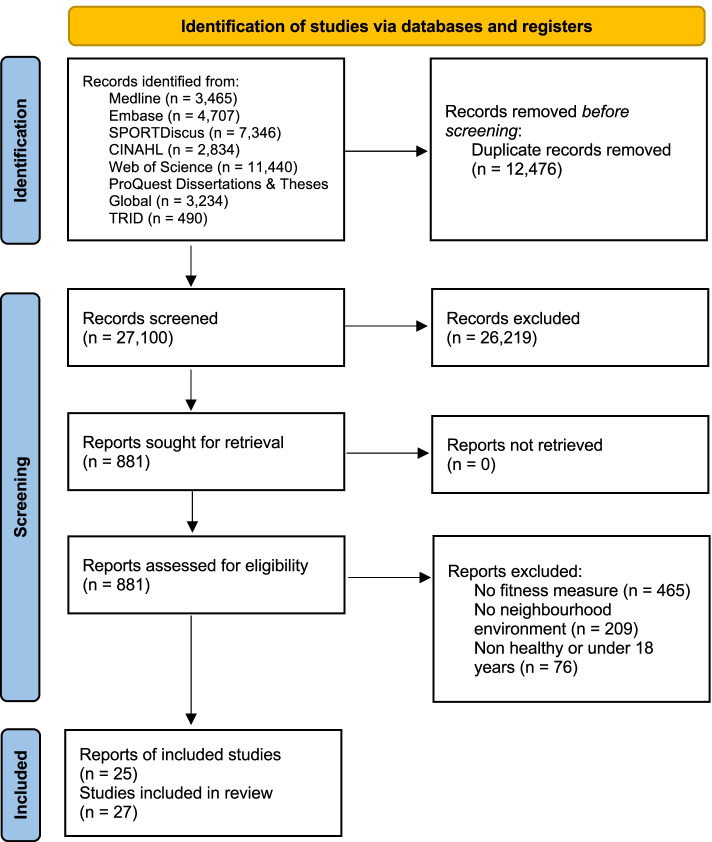
PRISMA flow diagram

After removal of duplicates, 27,100 records were screened. After reviewing 881 full-text reports, 25 reports were included [20–24, 29–31, 45–61]. Two of the included reports each included two different studies [24, 50] within the same paper and the findings of each study were reported separately; thus, 27 studies were included in the final narrative synthesis (Fig. 1).

### Characteristics of included studies

#### Study design

Table 1 shows the characteristics of the included studies. The majority (*n* = 21) of studies used cross-sectional designs [21, 23, 24, 29–31, 46–53, 55–61], with the remainder including cohort [20, 22, 24, 50, 54] (*n* = 5) or quasi-experimental [45] (*n* = 1) designs. Approximately half of studies (*n* = 14) were undertaken in the United States of America (USA) [22, 24, 29–31, 46, 48, 50, 51, 54, 56, 57] with the remainder undertaken in Japan [20, 49, 61] (*n* = 3), the United Kingdom (UK) [47, 58, 59] (*n* = 3), Canada [23, 55] (*n* = 2), France [45, 52] (*n* = 2), Brazil [53] (*n* = 1), China [21] (*n* = 1), and the Czech Republic [60] (*n* = 1). Sample sizes across studies ranged from 28 [45] to 419,562 [59]. Six studies included older adults (≥ 60 years) only [20, 22, 45, 49, 53, 61]. Six studies included female-only samples [45, 50, 51, 54, 60], while the remainder included multi-sexed/gendered samples [20–24, 29–31, 46–49, 52, 53, 55–59, 61].

**Table 1:** Summary of the methods for each included study (*n* = 25)

First author, year	Study, and sample design	Country	Sociodemographic characteristics	Neighbourhood definition	Built environment measures	Health-related fitness	Quality score^**a**^
Bailly, 2018 [45]	QE, probability	France	*n* = 28 Age range ≥ 65 yrs. Female = 100%	Administrative boundary	Obj: Urban infrastructure improvement (upgraded sidewalks, cross-roads, and central square, with signs indicating a pedestrian walking circuit)	Cardiorespiratory - 6-min walk test Morphological - Sit-and-Reach Test	8.5/9 (94%)
Brown, 2008 [22]	CH, probability	USA	*n* = 217 Age range ≥ 70 yrs. Female = 59%	Administrative boundary	Obj: University of Miami Built Environment Coding System (coded porches, stoops, and buildings built above grade)	Muscular - Hand grip dynamometry Cardiorespiratory - Gait speed	10/10 (100%)
Duchowny, 2020 [46]	CS, probability	USA	*n* = 11,277 Age range > 50 yrs. Female = 61%	A 20 min walk or about a mile (1600 m) around the participant’s home	SR: The physical neighbourhood disorder scale (presence of vandalism/graffiti, rubbish/litter, vacant/deserted homes, and crime)	Muscular - Hand grip dynamometry	7/7 (100%)
Ellaway, 2018 [47]	CS, probability	Scotland	*n* = 2040 Age range ≥ 18 yrs. Female = 55%	Participant identified perceived neighbourhood	SR: Neighbourhood quality (perceptions of, vandalism, litter and rubbish, assaults and muggings, disturbances by children or youngsters, smells and fumes, burglaries)	Morphological - BF% (BIA)	6/7 (86%)
Hoehner, 2011 [30]	CS, non-probability	USA	*n* = 16,543 Age range = 18-90 yrs. Female = 30%	Census block	Obj: Spatial (neighbourhood walkability at the block-group level, population density, housing type, median home age, and commuting patterns)	Cardiorespiratory - Maximal treadmill test (modified Balke protocol)	7/7 (100%)
Hoehner, 2013 [29]	CS, non-probability	USA	*n* = 8857 Age range = 18-90 yrs. Female = 29%	800 m polygon network buffer and a 1600 m radial buffer	Obj: Spatial (land use mix, intersection density, household density, vegetation, sidewalk coverage, and speed limits in an 800 m network polygon buffer and public and private exercise facilities, and parks in a 1600 m radial buffer)	Cardiorespiratory - Maximal treadmill test (modified Balke protocol)	7/7 (100%)
Johnson, 2006 [48]	CS, probability	USA	*n* = 61 Age range = University freshmen, Female = 54%	A 10 min walk or about a half mile (800 m) around the participant’s home	SR: NEWS (eight subscales, residential density, land use mix-diversity, land use mix-access, connectivity, walking/cycling facilities, aesthetics, traffic safety, and crime safety)	Morphological - BF% (DXA)	6/7 (86%)
Koohsari, 2020 [26, 49]	CS, probability	Japan	*n* = 314 Age range = 65-84 yrs. Female = 38%	800 and 1600 m road network buffer	Obj: GIS (population density, availability of destinations, intersection density, and distance to the nearest public transport station); Walk Score®	Muscular - Hand grip dynamometry Cardiorespiratory - Gait speed Motor - One legged stance test (eyes open); timed up-and-go	7/7 (100%)
Leach, 2013 [50]	Study 1: CH, probability Study 2: CS, probability	USA	Study 1: n = 30 Age range = 33.7-60.3 yrs. Female = 100% Study 2: *n* = 30 Age range = 39.0-66.0 yrs. Female = 100%	800 m buffer; census block-group level	Obj: PARs (walkability (PEDs), and traffic safety (average speed limit) in an 800 m buffer and crime safety (FBI Uniform Crime Report) at the census block-group level)	Cardiorespiratory - Submaximal treadmill test (Modified Balke protocol) Morphological - BF% (BIA)	Study 1: 10/10 (100%) Study 2: 7/7 (100%)
Lee, 2012 [51]	CS, non-probability	USA	*n* = 383 Age range = 25-60 yrs. Female = 100%	800 m Euclidean buffer	Obj: PEDs (pedestrian crossing aids, sidewalk traffic buffers, traffic control devices, number of path connections, number of travel lanes, posted speed limit, amenities, and safety and attractiveness of the walking and cycling environment)	Morphological - BF% (BIA)	7/7 (100%)
Lee, 2017 [24]	Study 1: CS, non- probability Study 2: CH, non-probability	USA	Study 1: *n* = 5435 Age range > 18 yrs. Female = 54% Study 2: *n* = 4010 Age range > 18 yrs. Female = 55%	Census block and census tract	Obj: Census block data (intersection density, greenspace, and recreation land); census tract (food stores)	Morphological - VAT and SAT measured by abdominal scans using an eight-slice multi-detector computed tomography	Study 1: 7/7 (100%) Study 2: 8.5/10
Lewin, 2014 [52]	CS, non-probability	France	*n* = 4078 Age range = 30-79 yrs. Female = 28%	1000 m network buffer	Obj: Spatial (density of population, density of destinations, density of three-way street intersections, the surface of green spaces, the number of supermarkets/hypermarkets, and proportion of fast-food restaurants among all restaurants)	Morphological - FMI FM% (BIA)	7/7 (100%)
McCormack, 2020 [23]	CS, probability	Canada	*n* = 592 Age range ≥ 18 Female = 67%	Administrative boundary	SR: PANES (dwelling types, destinations, transit, sidewalks, bicycle infrastructure, recreational facilities, crime, traffic, connectivity, and aesthetics); PPI (parks in the neighbourhood, park attractiveness, and park safety) Obj: Walk Score®	Muscular - SR: Perceived muscle strength Cardiorespiratory - SR: Perceived cardiorespiratory fitness Morphological - SR: Perceived flexibility	7/7 (100%)
Nascimento, 2018 [53]	CS, probability	Brazil	*n* = 1190 Age range ≥ 60 yrs. Female = 60%	Administrative boundary	Obj: Administrative data (green space and crime statistics)	Motor - Timed up-and-go test	7/7 (100%)
Nies, 2002 [54]	CH, NR	USA	*n* = 313 Age range = 30-60 yrs. Female = 100%	Administrative boundary	SR: Three questions (1) Does your neighborhood provide places for you to walk outdoors? 2) Does your neighborhood provide places to walk outdoors that are convenient for you to walk? 3) Do you have people in your neighborhood that can walk with you?)	Cardiorespiratory - Rockport one-mile walk test Morphological - BF% via circumference measures (waist, right thigh, and right calf)	6/10 (60%)
Okuyama, 2020 [20]	CH, probability	Japan	*n* = 2526 Age range > 60 yrs. Female = 60%	1000 m network buffer	Obj: GIS (hilliness, bus stop density, intersection density, residential density, and distance to community centers)	Muscular - Hand grip dynamometry Morphological - SMI (BIA)	8/10 (80%)
Petrella, 2008 [55]	CS, probability	Canada	*n* = 159 Age range = 48-92 yrs. Female = 55%	Neighbourhood not defined, network distance from home	Obj: GIS (shortest GIS-distance (km) to physical activity and eating facilities)	Cardiorespiratory - Maximal treadmill test (modified Naughton protocol)	5/7 (71%)
Pham, 2014 [56]	CS, probability	USA	*n* = 2881 Age range = 35-84 yrs. Female = 65%	Participant identified perceived neighbourhood	SR: Perceived neighbourhood safety (Likert scale - This neighborhood is safe from crime.)	Morphological - Total abdominal, subcutaneous, and visceral fat volume measured via computed tomography	6/7 (86%)
Rodriguez, 2003 [ 57 ]	CS, non-probability	USA	*n* = 206 Age range = College students Female = 44%	Participant identified perceived neighbourhood	SR: Perceived Environments Related to Physical Activity questionnaire (1) home environment, 2) neighborhood environment, and 3) convenient facilities)	Cardiorespiratory - SR: $\dot{V}$ O_2_ max estimated using the Jackson non-exercise model	7/7 (100%)
Sarkar, 2017a [58]	CS, non-probability	UK	*n* = 419,562 Age range = 37-73 yrs. Female = 56%	1000 m and 800 m street network buffer	Obj: UKBUMP (residential, land use, and public transport density - 1 km street network buffer; street-level movement density - 800 m street network buffer; Townsend deprivation score)	Morphological - BF% (BIA)	7/7 (100%)
Sarkar, 2017b [59]	CS, non-probability	UK	*n* = 333,183 Age range = 37-73 yrs. Female = 55%	500 m radial buffer	Obj: UKBUMP (residential greenness - NDVI)	Morphological - Whole body fat (BIA)	7/7 (100%)
Shaffer, 2017 [31]	CS, non-probability	USA	*n* = 445 Age range = University students Female = 47%	A 10 min walk or about a half mile (800 m) around the participant’s home	SR: NEWS (perceptions of sidewalks, traffic, crime and seeing others active in their neighborhood); living complex access to individual PA resources (“individuals reported (yes/no) if their apartment complex offered resources for PA (e.g. weight room, cardio equipment, pool, etc.), which was summed (range 0-12)”); Indication if others are active in their building and if their building provides PA resources (Likert scale))	Muscular – Timed push-up and maximum repetition curl-up test Cardiorespiratory - YMCA Submaximal Cycle Ergometer test Morphological - BF% (BIA)	6/7 (86%)
Sofkova, 2013 [60]	CS, non-probability	Czech Republic	*n* = 167 Age range = 20-60 yrs. Female = 100%	A 10 min walk or about a half mile (800 m) around the participant’s home	SR: NEWS-A (residential density, land use mix diversity, access to services, street connectivity, walking and cycling facilities, aesthetics, traffic safety, and crime safety)	Morphological – FFM, BF% (BIA)	5.5/7 (79%)
Soma, 2017 [61]	CS, probability	Japan	*n* = 509 Age range = 65-86 yrs. Female = 53%	1000 m line-based road network buffer	Obj: GIS (population density, number of daily life-related destinations, community centres, medical facilities, and recreational facilities)	Muscular - Hand grip dynamometry, five repetition sit-to-stand Cardiorespiratory - Habitual walking speed Motor - Timed up-and-go	7/7 (100%)
Sun, 2020 [21]	CS, non-probability	China	*n* = 728 Age range ≥ 18 yrs. Female = 60%	A 10 min walk or about a half mile (800 m) around the participant’s home	SR: NEWS-A (residential density, land use mix diversity, access to services, street connectivity, walking and cycling facilities, aesthetics, traffic safety, and crime safety)	Muscular - Hand grip dynamometry, timed curl-up test Motor - One-foot standing test Morphological – BF% (BIA), Sit-and-Reach test	7/7 (100%)

All reported health-related fitness measures are objectively-measured unless otherwise stated

*BF%* Body fat percentage, *BIA* Bioelectrical impedance, *CH* Cohort, *CS* Cross-sectional, *DXA* Dual-energy x-ray absorptiometry, *FMI* Fat Mass Index, *FM%* Fat mass percent, *GIS* Geographic information systems, *NDVI* Normalized difference vegetation index, *NEWS* Neighborhood Environment Walkability Scale, *NEWS-A* Abbreviated Neighborhood Environment Walkability Scale, *NR* Not reported, *Obj* Objectively measured, *PANES* Physical Activity Measurement Scale, *PEDS* Pedestrian Environmental Data Scan, *PPI* Park Perceptions Index, *QE* Quasi Experimental, *SAT* Subcutaneous adipose tissue, *SMI* Skeletal muscle mass index, *SR* Self-reported, *UK* United Kingdom, *UKBUMP* UK Biobank Urban Morphometric Platform, *USA* United States of America, *VAT* Visceral adipose tissue

^a^Quality scored used the JBI checklist for cross-sectional, cohort, or non-randomized experimental studies

#### Built environment measures

Among the 17 studies that included an objective measure of the built environment, neighbourhood geography was either operationalized using ego-centric spatially-defined buffers (or polygons) or distances around or from participants geo-located residential households [20, 29, 49–52, 55, 58, 59, 61] (*n* = 11) or by administrative boundaries [22, 24, 30, 45, 50, 53] (*n* = 6). The size of the buffers used ranged from 500m [58] to 1600 m [29, 49], with 800m [29, 49–51, 59] (*n* = 6) being the most commonly used definition. Among the 10 studies that included a measure of self-reported built environment, four used the Neighborhood Environment Walkability Scale (NEWS) [21, 48, 59, 60], two studies captured perceptions about places in the neighbourhood to be active [54, 57], one study used the Physical Activity Neighborhood Environment Scale (PANES) [23], one study each captured perceived neighbourhood disorder [46], perceived neighbourhood quality [47], and perceived safety [56].

The most common neighbourhood built environment characteristics measured included *desirability* (*n* = 13) [21, 22, 24, 29, 31, 47, 48, 50, 51, 53, 56, 58, 60], followed by *diversity* (*n* = 12) [21, 24, 29, 31, 48–51, 54, 57, 60, 61], *design* (*n* = 10) [20, 21, 24, 29, 31, 48–51, 60] and *composite or other features* (*n* = 10) [21, 23, 30, 31, 45, 46, 49, 50, 57, 60], *density* (*n* = 9) [20, 21, 29, 48, 49, 52, 59–61], *destination proximity* (*n* = 6) [20, 29, 48, 54, 55, 60] and *transit* (*n* = 2) [20, 49] features. No study measured *distributed* features. The most common built environment elements measured under *diversity* features included the availability or presence of specific destination types [20, 21, 24, 29, 31, 48, 49, 51, 52, 55, 57, 59–61]. Street connectivity and residential density were the most common built environment elements under *design* and *density*, respectively [20, 21, 24, 29, 31, 48, 49, 51, 52, 59–61]. For *desirability* features, both greenspace and perceived neighbourhood aesthetics were the most common elements measured [21, 24, 29, 31, 48, 51–53, 58, 60]. *Desirability* also included measures of safety [21, 31, 48, 50, 51, 53, 56, 60]. Walkability was the most common element under *composite or other features* [21, 23, 30, 31, 49, 60].

#### Health-related fitness measures

With the exception of two studies [23, 57], all health-related fitness measurements were objectively measured [20–22, 24, 29–31, 45–56, 58–61]. There was a total of eight studies that included at least one measure of muscular fitness [20–23, 31, 46, 49, 61]. Six studies included grip strength [20–22, 46, 49, 61], while a timed curl-up test [21], a maximal repetition curl up test (up to 75 repetitions) [31], timed push-up [31], a 5-repetion sit-to-stand test [61], and self-reported muscular strength were each reported in individual studies [23]. Among the 12 studies measuring cardiorespiratory fitness, six used an estimation of $\dot{V}{O}_2\ \mathit{\max}$ [29–31, 50, 55, 57], three included habitual walking speed [22, 49, 61], two included timed distance tests [45, 54], and one included self-reported cardiorespiratory fitness [23]. Among the four studies measuring motor fitness, three used the Timed Up-and-Go test [49, 53, 61] and two used a timed one-foot standing test [21, 49]. There was a total of 16 studies that included at least one measure of morphological fitness [20, 21, 23, 24, 31, 45, 47, 48, 50–52, 54, 56, 58–60]. Fourteen studies included measurements of body composition [20, 21, 24, 31, 47, 48, 50–52, 54, 56, 58–60], two studies used Sit-and-Reach tests [21, 45], and one reported perceived flexibility [23].

### Study quality

Most studies (*n* = 17) were assessed to have the highest methodological quality score possible for their respective study design (cross-sectional, cohort or quasi-experiments). Cross-sectional studies of lower methodological quality tended to inadequately describe the sample design and setting and the reliability or validity of the built environment measures, and/or they did not control for confounders [31, 47, 48, 55, 56, 60]. Quasi-experiments of lower quality tended to provide unclear descriptions of their follow-up data collection [45]. Lower quality cohort studies tended to inadequately describe the follow-up data collection or the reliability or validity of the built environment measures, and/or they did not control for confounders [20, 24, 54].

### Adjustment for physical activity

Out of the 27 studies, eleven (40.7%) adjusted for physical activity. Physical activity was adjusted for in four of the eight studies that assessed muscular fitness [20, 21, 23, 61], in five of the twelve studies that assessed cardiorespiratory fitness [23, 29, 30, 50, 61], in two of the four studies that assessed motor fitness [21, 61], and in the eight of the sixteen studies that assessed morphological fitness [20, 21, 23, 47, 50, 52, 56, 59].

### Associations between the neighbourhood built environment and health-related fitness

#### Muscular fitness

Table 2 summarizes the associations between neighbourhood built features and muscular fitness. Excluding *distributed* features, all other built features were examined in relation to muscular fitness. Across these features, all but two studies found either positive or null associations with muscular fitness.

**Table 2:** Associations between neighbourhood built environment features and muscular fitness (*n* = 8 studies)

Author, year	Design	Density	Transit	Destination proximity	Diversity	Desirability	Distributed	Composite or other
Brown, 2008 [22]						+		
Duchowny, 2020 [46]								–
Koohsari, 2020 [26, 49]	=	=	=		=			=
Shaffer, 2017 [31]	=				=	=		=
McCormack, 2020^a^ [23]								+, =
Okuyama, 2020^a^ [20]	+, =	=	-, =	+, =				
Soma, 2017^a^ [61]		+, =			+, =			
Sun, 2020^a^ [21]	=	+, =			=	+, =		=
Total associations^b^	+^1^, =^4^	+^2^, =^4^	-^1^, =^2^	+^1^, =^1^	+^1^, =^4^	+^2^, =^2^		+^1^, −^1^, =^4^

+: any statistically significant positive association

-: any statistically significant negative association

=: any non-statistically significant association

^a^adjustment for self-reported physical activity

^b^Superscript indicates total number of studies finding positive, negative or null associations

Self-reported street connectivity was positively associated with curl-up performance in a cross-sectional study of Chinese women [21] while topography (i.e., slope steepness) was positively associated with grip strength in a cohort of Japanese males [20]. No studies found neighbourhood safety to be associated with muscular fitness [21, 31]. Brown et al. [22] found positive associations between neighbourhood architecture and grip strength, while Sun et al. [21] found positive associations between self-reported neighbourhood aesthetics and curl-up performance in males. In a cross-section of older Japanese adults, having more utilitarian destinations (men and women), recreational facilities (men and women), and medical facilities (men only) in the neighbourhood was associated with better performance in the Sit-to-Stand test [61]. Moreover, among women, a greater number of neighbourhood utilitarian destinations and medical facilities was positively associated with grip strength [61]. Among a cohort of older Japanese women, neighbourhood bus stop density was negatively associated with grip strength [20]. Composite features were also associated with muscular fitness. In a cross-sectional sample of adults from the USA, neighbourhood physical disorder (vandalism/graffiti, rubbish/litter, vacant/deserted homes, and crime) was negatively associated with grip strength, although the study did not adjust for physical activity [46]. In a cross-sectional study of Canadian adults, self-reported neighbourhood walkability was positively associated with perceived muscle strength [23].

Adjusting for physical activity, there were five positive, one negative and ten null associations between built environment features and muscular fitness. Although attenuated, after adjustment for self-reported frequency of achieving sufficient MVPA (≥30 minutes/day) in the past week and self-reported days of resistance training in a usual week, perceived overall neighbourhood walkability was still positively associated perceived muscular fitness in a Canadian population [23]. After adjusting for self-reported physically activity habit (yes/no), land slope remained positively associated and bus stop density negatively associated, with objectively measured grip strength in Japanese adults [20]. In another Japanese sample, the number of neighbourhood destinations were positively associated with objectively measured grip strength after adjusting for self-reported total (i.e., occupation, household, and leisure) physical activity [61]. In a sample of Chinese adults, after adjustment for self-reported total MVPA (i.e., weekly MET-minutes), for men perceived neighbourhood aesthetics and for women street connectivity, were positively associated with curl-up performance [21].

#### Cardiorespiratory fitness

Table 3 summarizes the associations between the neighbourhood built environment and cardiorespiratory fitness. Excluding *distributed* features, all other built features were examined in relation to cardiorespiratory fitness. Among these, *transit* features were not associated with cardiorespiratory fitness, while the other features were found to have positive or null associations with cardiorespiratory fitness.

**Table 3:** Associations between neighbourhood built environment features and cardiorespiratory fitness (*n* = 12 studies)

Author, year	Design	Density	Transit	Destination proximity	Diversity	Desirability	Distributed	Composite or other
Bailly, 2018 [45]								+
Brown, 2008 [22]						+		
Koohsari, 2020 [26, 49]	=	=	=		=			=
Nies, 2002 [54]				+	+			
Petrella, 2008 [55]				+, =				
Rodriquez, 2003					+			=
Shaffer, 2017 [31]	=				=	=		=
Hoehner, 2011^a^ [30]								+
Hoehner, 2013^a^ [29]	+	=		=	+, =	+, =		
Leach, 2013^a^ [50]						=		
McCormack, 2020^a^ [23]								+, =
Soma, 2017^a^ [61]		+			+, =			
Total associations^b^	+^1^, =^2^	+^1^, =^2^	=^1^	+^2^, =^2^	+^4^, =^4^	+^2^, =^3^		+^3^, =^4^

+: any statistically significant positive association

-: any statistically significant negative association

=: any non-statistically significant association

^a^adjustment for self-reported physical activity

^b^Superscript indicates total number of studies finding positive, negative or null associations

A cross-sectional analysis of American adults, found that intersection density was positively associated with maximal metabolic equivalent of task (MET) values [29]. In older Japanese adults, population density was positively associated with an increased walking speed [61]. No studies found neighbourhood safety to be associated with cardiorespiratory fitness [31, 49, 50]. A cross-sectional study found that a front facing architecture type (including porches, stoops, and buildings built above grade) was positively associated with gait speed in a cohort of older Hispanic Americans [22]. Further, Hoehner et al. [29] found positive cross-sectional associations between a greater proportion of vegetation in the neighbourhood and maximal METs. In cross-sectional associations, the number of private exercise facilities, and community centres were positively associated with maximal METs and habitual walking speed, in samples of American [29] and Japanese [61] adults, respectively. In three separate cross-sectional samples, distance to dance studios and baseball diamonds was positively associated with $\dot{V}{O}_2\ \mathit{\max}$ in Canadian adults [55], perception of places to walk in the neighbourhood was positively correlated with 1-mile walk scores in American women [54], and perceptions of convenient neighbourhood facilities was positively associated with estimated $\dot{V}{O}_2\ \mathit{\max}$ in American adults [57]. Composite built environment associations with cardiorespiratory fitness included an intervention of older French women, where an improved urban environment consisting of a pedestrian circuit, improved roadway accessibility and rehabilitation of a central square, was positively associated with 6-minute walk scores [45]. In a cross-section of American adults, more walkable neighbourhoods, and non-auto commuting neighbourhoods, were positively associated with maximal METs for males and females, and males only, respectively [30]. In a cross-section of Canadian adults, self-reported neighbourhood walkability was positively associated with perceived cardiorespiratory fitness [23].

Among studies that adjusted for physical activity, there were seven positive and nine null associations between built environment features and cardiorespiratory fitness. Although attenuated, after adjusting for self-reported weekly MET-minutes of outdoor physical activity, traditional core neighbourhoods remained positively associated with maximal metabolic equivalents obtained through a treadmill test in American adults [19]. In another sample of American adults, after adjustment for self-reported weekly MET-minutes of MVPA, associations between and intersection density and maximal MET were no longer statistically significant; however, associations between greenspace (positive), the number of exercise facilities in the neighbourhood (positive), and distance to the closest city center (negative) remained significant [29]. Moreover, after adjustment for self-reported MVPA (≥30 minutes/day) in the past week and self-reported days of resistance training in a usual week, perceived overall neighbourhood walkability remained positively associated with self-reported cardiorespiratory fitness in a sample of Canadian adults [23]. Further, in a sample of Japanese older adults, population density and the number of community centers in the neighbourhood remained positively associated with walking speed after adjusting for total (i.e. occupational, household and leisure) self-reported physical activity measured using the Physical Activity Scale for the Elderly [61].

#### Motor fitness

Table 4 summarizes the associations between the neighbourhood built environment and motor fitness. Excluding *distributed* and *destination* features proximity, all other built features were examined in relation to motor fitness. Across these features, *transit*, *desirability*, and *composite or other features* were not found to be associated with motor fitness while *design*, *density*, and *diversity* were found to be positively or not associated with motor fitness.

**Table 4:** Associations between neighbourhood built environment features and motor fitness (*n* = 4 studies)

Author, year	Design	Density	Transit	Destination proximity	Diversity	Desirability	Distributed	Composite or other
Koohsari, 2020 [26, 49]	+, =	+, =	=		+, =			=
Nascimento, 2018 [53]						=		
Soma, 2017^a^ [61]		=			=			
Sun, 2020^a^ [21]	=	=			=	=		=
Total associations^b^	+^1^, =^2^	+^1^, =^3^	=^1^		+^1^, =^3^	=^2^		=^2^

+: any statistically significant positive association

-: any statistically significant negative association

=: any non-statistically significant association

^a^adjustment for self-reported physical activity

^b^Superscript indicates total number of studies finding positive, negative or null associations

A cross-sectional study of older Japanese males, population density within a 1600 m neighbourhood buffer, and intersection density within an 800 m neighbourhood buffer was positively associated with timed one-legged stance scores (with eyes open) [49]. Although the study did not adjust for physical activity. There were no associations between safety [21, 53] or aesthetics [21, 53] of the neighbourhood built environment and motor fitness. In the same sample of older Japanese males, availability of destinations within the 1600 m neighbourhood buffer were positively associated with timed one-legged stance scores (with eyes open) [49]. There were no associations between composite built environment measures and motor fitness [21, 49].

Associations between the built environment and motor fitness were not statistically significant after adjustment for physical activity [21, 61].

#### Morphological fitness

Table 5 summarizes the associations between the neighbourhood built environment and morphological fitness. Excluding distributed features, all other built features were examined in relation to morphological fitness. Among these features, for morphological fitness negative associations were found for *transit*, null associations found for *destinations*, negative and null associations found for *design*, and negative, null, and positive associations found for density, *diversity*, *desirability*, and *composite or other features*.

**Table 5:** Associations between neighbourhood built environment features and morphological fitness (*n* = 16 studies)

Author, year	Design	Density	Transit	Destination proximity	Diversity	Desirability	Distributed	Composite or other
Bailly, 2018 [45]								+
Johnson, 2006 [48]	=	=		=	+	=		
Lee, 2012 [51]	=				–	=		
Lee, 2017 [24]	-, =				-, =	+, =		
Nies, 2002 [54]				=	=			
Sarkar, 2017b [59]						–		
Shaffer, 2017 [31]	=				=	=		=
Sofkova, 2013 [60]	=	=		=	=	=		-, =
Ellaway, 2018^a^ [47]						+		
Leach, 2013^a^ [50]	=				=	=		=
Lewin, 2014^a^ [52]		-, =						
McCormack, 2020^a^ [23]								+, =
Okuyama, 2020^a^ [20]	=	=	–	=				
Pham, 2014^a^ [56]						-, =		
Sarkar, 2017a^a^ [58]		+, −, =						
Sun, 2020^a^ [21]	=	=			-, =	-, =		=
Total associations^b^	-^1^, =^8^	+^1^, −^2^, =^6^	-^1^	=^4^	+^1^, −^3^, =^6^	+^2^, −^2^, =^8^		+^2^, −^1^, =^5^

+: any statistically significant positive association

-: any statistically significant negative association

=: any non-statistically significant association

^a^adjustment for self-reported physical activity

^b^Superscript indicates total number of studies finding positive, negative or null associations

A cohort study of American adults found intersection density negatively associated with changes in visceral adipose tissue [24]. A cross-sectional study of French adults found residential density negatively associated with both fat mass index and percent fat mass in males [52]. A cross-sectional study in the UK found a curvilinear relationship between residential density and body fat [59]. Specifically, residential density was positively associated with body fat ≤1800 units per km [2] then negatively associated with body fat > 1800 units per km^2^ [59]. Perceptions of neighbourhood safety were negatively associated with visceral adipose tissue in a cross-section of African American females [56]. A cross-sectional study of Chinese adults found that perceived pedestrian and traffic safety was negatively associated with sit-and-reach scores in males [21]. Lee et al. [24] found that greenspace was positively associated with change in visceral adipose tissue in a cohort of American adults. Conversely, in a cross-sectional sample of UK adults, residential greenness was negatively associated with body fat [58].

A cross-sectional study of American university students found perceptions of access to destinations was negatively associated with body fat percentage in males [48]. A cross-sectional study of ethnic minority American women found objectively measured neighbourhood amenities were negatively associated with body fat percentage [51]. Lee et al. [24] found that total food stores, full-service restaurants, fast food restaurants, supermarkets, and convenience stores was negatively associated with a change in visceral adipose tissue. Bus stop density was negatively associated with skeletal mass index in a cohort of Japanese males [20]. Perceptions of neighbourhood access to services and land use mix diversity were negatively associated with sit-and-reach scores in Chinese males [21].

For composite features, an intervention including older French women found an improved urban environment consisting of a pedestrian circuit, improved roadway accessibility and rehabilitation of a central square, to be positively associated with sit-and-reach test scores [45]. In cross-sectional analyses of three different cohorts, Ellaway et al. [47] found that an index of perceived neighbourhood problems (vandalism, litter, crime, youth disorderly conduct, and foul odor) was positively associated with change in body fat percentage over time. In Canadian adults, McCormack, et al. [23] found that perceptions of neighbourhood walkability and a park quality score were positively associated with perceived flexibility.

Adjusting for physical activity there were five positive, four negative and fourteen null associations with morphological fitness. After adjusting for the self-reported number of days per week performing vigorous exercise (≥20 minutes continuous), body fat percent remained positively associated with perceived neighbourhood problems [47]. Further, after adjusting for different levels of activity in varying occupations, residential density was inversely associated with fat mass index and percent fat mass in males [52]. Moreover, after adjustment for self-reported weekly MVPA (≥30 minutes/day) and self-reported days of resistance training in a usual week, perceived overall neighbourhood walkability remained positively associated with self-reported flexibility among Canadian adults [23]. Among Japanese older males, bus stop density was negatively associated with skeletal muscle index after adjusting self-reported physically active habit [20]. Adjusting for physical activity measured via an active living index (i.e., frequency and duration of physical activities minus frequency and duration of sedentary behavior), neighbourhood safety was positively associated with visceral and total adipose tissue in premenopausal women [56]. In in a large UK sample, after adjustment for self-reported physical activity (weekly MET hours), population density was found to have a non-linear association with objectively measured whole body fat [59]. Among Chinese males, perceived neighbourhood destinations and safety was negatively associated with sit and reach performance, after adjustment for self-reported MVPA in weekly MET minutes [21].

## Discussion

We found 27 different studies that estimated the relationship between the neighbourhood built environment and health-related fitness. The reviewed evidence suggests that specific built environment features are more often than not to have either a positive or no association with health-related fitness. Moreover, this evidence suggests that associations between the built environment and health-related fitness persist, albeit attenuated, after controlling for physical activity. Using the updated built environment framework by Giles-Corti et al. [19] we found specific built characteristics associated with *design*, *density*, *diversity*, and *desirability* features to be the most commonly studied; while no studies examined built characteristics associated with *distributed* features.

The most common component of health-related fitness investigated was morphological fitness, with an emphasis on body composition. The negative associations between the built environment and body composition found in our review tend to support findings from previous reviews summarizing evidence related to built environment and weight outcomes [42, 43, 62]. Our findings suggest that having multiple, easily accessible destinations within a neighbourhood may favorably influence body composition. This result is congruent with longitudinal findings suggesting that having multiple, easily accessible destinations within a neighbourhood is linked to favorable changes in physical activity behaviour [17, 63].

The second most common association between the neighbourhood built environment and health-related fitness category was with cardiorespiratory fitness, and in general, measurements of estimated maximal aerobic capacity. Given the link between physical activity and cardiorespiratory fitness, our findings tend to support those that have been found previously between the built environment and physical activity [17, 63]. Similar to associations between the built environment and morphological fitness, having multiple destinations within a neighbourhood that are easily accessible was associated with favorable cardiorespiratory fitness. There are multiple lines of evidence, including cross-sectional [17, 44], longitudinal [63], and natural experiments [63], indicating favorable changes in physical activity behaviour with improvements in neighbourhood destinations.

Overall, the results of our review indicate that physical activity likely mediates, at least partially, associations between the neighbourhood built environment and health-related fitness. There are numerous explanations as to the mechanisms explaining how the built environment might be positively associated with health-related fitness. For example, carrying heavy loads in the hands is related to forearm musculature activity [64] and muscular fitness, therefore, in areas with a higher land-use mix, residents may walk to complete daily errands and carry items back to their residence, which may slow impairments to activities of daily living [65]. Recreational facilities located within walking distance of home, where resistance or aerobic training might be performed, may explain positive associations between the neighbourhood built environment and cardiorespiratory and muscular fitness. Increases in motor fitness has been shown through proprioceptive exercises such as wobble boards or unstable activities [66]. Speculatively, neighbourhoods with high population density, street connectivity, and land use mix, may provide opportunities to manoeuvre around obstacles (i.e., people, benches, traffic bollards etc.), which may emulate some movements undertaken during structured proprioceptive exercises. Among older adults, more frequent falls, which are associated with motor fitness [67], have been found in peripheral areas compared with city areas [68]. There is also consistent evidence demonstrating associations between neighbourhood walkability and walking [69, 70], which subsequently could result in improved cardiovascular [71], and morphological fitness [72].

However, other pathways may exist linking built environment with fitness that are not mediated by physical activity. For example, traffic density, which is associated with the built environment (e.g., air pollution) [19], can have detrimental effects on cardiorespiratory fitness [73, 74]. Diet, which is associated with morphological fitness [75], is also associated with the built environment (e.g., proximity and availability of fast food restaurants, supermarkets, and convenience stores) [62, 76].

Our findings suggest that the built environment may have effects on health-related fitness independent of physical activity. However, studies adjusting for physical activity did so using self-reported physical activity, which may not accurately capture the total volume nor intensities of physical activity undertaken. Moreover, among these studies few included measures of transport-related physical activity that may be more strongly associated with the built environment [77].

### Strengths and weaknesses

A strength of our review is the overall breadth of included exposures, outcomes, and study designs. Capturing multiple components of health-related fitness allowed for a broader scope of the literature to be evaluated and to better theorize the multiple ways in which the built environment might impact health-related fitness. However, our broader research objective may have contributed to heterogeneous sample of studies included in our review which together with their dissimilar sample designs and methods, limited our ability to conduct a meta-analysis.

Limitations common in the literature exploring the relationships between physical activity and the neighbourhood built environment were also present in studies included this review. The lack of control for residential exposure time [78] and residential self-selection [79] was pronounced in our summary. In our review, we only found two of studies that controlled for length of residential exposure time [49, 50]. The lack of control for residential self-selection is also an important variable in neighbourhood built environment research; however, we found no studies controlling for this potential confounder. This confounder is potential important because individual who undertake physical activity for the main purpose of improving or maintaining their health-related fitness may choose to reside in neighbourhoods that have built features that support desired physical activities (e.g., access to parks, pathways, recreational facilities). Speculatively, not adjusting for residential self-selection could lead to over-estimates of the association between the built environment and health-related fitness, especially in cross-sectional studies [79]. Further, our study quality tools assessed the quality of reporting limiting our ability to assess bias. Moreover, as many of the identified studies were cross-sectional in design assessment of causality is limited.

### Future directions

Evidence suggests that the built environment, through its potential influence on physical activity, is associated with a range of health outcomes such as cardiovascular disease, overweight and obesity, and type 2 diabetes [36]. Findings from our review suggest that health-related fitness is another important factor that should be considered when exploring the role of the built environment in supporting health, especially given its relationships both with physical activity [5] and chronic disease [6]. Future research is needed to examine the causal pathways between the built environment and health-related fitness, not only via physical activity but also other potential mediators (e.g., sedentary behaviour, air pollution). To generate rigorous evidence for informing urban design and public health policy and interventions, this future research should include longitudinal, experimental, and quasi-experimental study designs that incorporate objective measures of the built environment, health-related fitness, and physical activity (and other mediators).

## Conclusion

The neighbourhood built environment appears to be associated with all components of health-related fitness (i.e., muscular, cardiorespiratory, motor, and morphological fitness). Somewhat expectedly, our findings of the built environment-health-related fitness relationship tend to mirror the built environment-physical activity evidence in that a more supportive neighbourhood built environments can support higher levels of physical activity [17, 63]. However, while physical activity might be an important mediator between the built environment and health-related fitness, our findings suggest there are potentially behaviours or factors other than physical activity that might explain some of the association between the neighbourhood built environment and health-related fitness. The relationship between the neighbourhood built environment and health-related fitness may be a promising area to improve public health. However, to make firm policy, practice, and design recommendations, future research on the associations between the neighbourhood environment and health-related fitness that controls for important confounders is needed (e.g., objectively-measured physical activity, resistance training, sedentary behaviour, diet, neighbourhood exposure, and residential self-selection).

**Box 1:** Glossary of key terms

**Term**	**Definition**
Built environment	The man-made structures, amenities, features, and facilities in which people live, work, and undertake leisure.
Design	Design refers to the connectivity, permeability, and layout of neighbourhood streets.
Density	Density refers to the clustering and amount of residential accommodations in an area that allow local business and public transportation to be supported.
Transit	Transit refers to the availability, accessibility, and location of public transportation.
Destination proximity	Destination proximity refers to the accessibility and location of local amenities or points of interest.
Diversity	Diversity refers to residential areas that a built with different types of housing and integrated with commercial, public, and recreational facilities and/or opportunities.
Desirability	Desirability refers to neighbourhoods that are safe, aesthetically pleasing, and comfortable.
Distributed	Distributed refers to neighbourhoods that have resources that promote equity for its residence.
Walkability	Walkability is a combination of two or more individual built environment characteristics or features that together support being physically active.
Health-related fitness	Health-related fitness is a combination of characteristic that result in a state of being that is associated with vigour and a decreased risk of morbidity and mortality that result from a sedentary lifestyle. The health-related fitness of an individual can be categorized into five components (muscular, cardiorespiratory, motor, morphological, and metabolic).
Muscular fitness	Muscular fitness is the combination of muscular strength and muscular endurance. Muscular strength is the ability of the musculature to exert an external force. Muscular endurance is the ability of the musculature to exert continued or repetitious force or contraction.
Cardiorespiratory fitness	Cardiorespiratory fitness refers to the ability of the circulatory and respiratory systems to undertake sustained and/or maximal activity and the ability to efficiently recover after being physical active.
Motor fitness	Motor fitness refers to proprioceptive abilities such as balance, agility, and coordination. Balance relates to maintaining equilibrium while moving or stationary. Agility refers to the ability to change positions with speed and accuracy. Coordination refers to using a combination of senses such as sight and hearing along with muscular movement.
Morphological fitness	Morphological fitness refers to overall body measurement such as height, weight, and body composition, muscle mass, adiposity, and bone density, as well as flexibility. Flexibility relates to the range of motion available at a joint.
Metabolic fitness	Metabolic fitness refers to biomarkers and processes that may influence health such as glucose tolerance, insulin sensitivity, and blood lipid concentrations.

Built environment definitions were adapted from Giles-Corti et al. [18] and Giles-Corti et al. [19]

Health-related fitness definitions were adapted from Caspersen et al. [80], Shephard [8], and Vanhees et al. [6]

## Supplementary Information

**Additional file 1: S1.** PRISMA checklist. **S2.** Full search strategies

## Data Availability

The datasets used and/or analysed during the current study are available from the corresponding author on reasonable request.

## Abbreviations

BMI: Body mass index
PRISMA: Preferred Reporting Items for Systematic Reviews and Meta-Analyses
JBI: Joanna Briggs Institute
MET: Metabolic equivalent of task
USA: United States of America
UK: United Kingdom
NEWS: Neighborhood Environment Walkability Scale
PANES: Physical Activity Neighborhood Environment Scale
MVPA: Moderate-to-vigorous physical activity
