# Supplementary material for: The neighbourhood built environment and health-related fitness: a narrative systematic review
Source: Int J Behav Nutr Phys Act. 2022 Sep 24;19:124. doi: 10.1186/s12966-022-01359-0 (PMC9509561; doi:10.1186/s12966-022-01359-0)
Supplement: Supplementary file 1 — Additional file 1: S1. PRISMA checklist. S2. Full search strategies [file 12966_2022_1359_MOESM1_ESM.docx]

Supplementary Material

S1. PRISMA checklist

| **Section and Topic** | **Item #** | **Checklist item** | **Location where item is reported** |
| --- | --- | --- | --- |
| **TITLE** | | |  |
| Title | 1 | Identify the report as a systematic review. | Page 1 |
| **ABSTRACT** | | |  |
| Abstract | 2 | See the PRISMA 2020 for Abstracts checklist. | Page 2-3 |
| **INTRODUCTION** | | |  |
| Rationale | 3 | Describe the rationale for the review in the context of existing knowledge. | Page 4-6 |
| Objectives | 4 | Provide an explicit statement of the objective(s) or question(s) the review addresses. | Page 6 |
| **METHODS** | | |  |
| Eligibility criteria | 5 | Specify the inclusion and exclusion criteria for the review and how studies were grouped for the syntheses. | Page 7 |
| Information sources | 6 | Specify all databases, registers, websites, organisations, reference lists and other sources searched or consulted to identify studies. Specify the date when each source was last searched or consulted. | Page 6-7 |
| Search strategy | 7 | Present the full search strategies for all databases, registers and websites, including any filters and limits used. | Supplementary Material |
| Selection process | 8 | Specify the methods used to decide whether a study met the inclusion criteria of the review, including how many reviewers screened each record and each report retrieved, whether they worked independently, and if applicable, details of automation tools used in the process. | Page 6 |
| Data collection process | 9 | Specify the methods used to collect data from reports, including how many reviewers collected data from each report, whether they worked independently, any processes for obtaining or confirming data from study investigators, and if applicable, details of automation tools used in the process. | Page 6 |
| Data items | 10a | List and define all outcomes for which data were sought. Specify whether all results that were compatible with each outcome domain in each study were sought (e.g. for all measures, time points, analyses), and if not, the methods used to decide which results to collect. | Page 8 |
|  | 10b | List and define all other variables for which data were sought (e.g. participant and intervention characteristics, funding sources). Describe any assumptions made about any missing or unclear information. | Page 8 |
| Study risk of bias assessment | 11 | Specify the methods used to assess risk of bias in the included studies, including details of the tool(s) used, how many reviewers assessed each study and whether they worked independently, and if applicable, details of automation tools used in the process. | Page 8-9 |
| Effect measures | 12 | Specify for each outcome the effect measure(s) (e.g. risk ratio, mean difference) used in the synthesis or presentation of results. | NA: Narrative  Page 9 |
| Synthesis methods | 13a | Describe the processes used to decide which studies were eligible for each synthesis (e.g. tabulating the study intervention characteristics and comparing against the planned groups for each synthesis (item #5)). | Page 9 |
|  | 13b | Describe any methods required to prepare the data for presentation or synthesis, such as handling of missing summary statistics, or data conversions. | Page 9 |
|  | 13c | Describe any methods used to tabulate or visually display results of individual studies and syntheses. | Page 9; Table 1 |
|  | 13d | Describe any methods used to synthesize results and provide a rationale for the choice(s). If meta-analysis was performed, describe the model(s), method(s) to identify the presence and extent of statistical heterogeneity, and software package(s) used. | Page 9  NA: No Meta |
|  | 13e | Describe any methods used to explore possible causes of heterogeneity among study results (e.g. subgroup analysis, meta-regression). | NA: No Meta |
|  | 13f | Describe any sensitivity analyses conducted to assess robustness of the synthesized results. | NA: No Meta |
| Reporting bias assessment | 14 | Describe any methods used to assess risk of bias due to missing results in a synthesis (arising from reporting biases). | NA: No Meta |
| Certainty assessment | 15 | Describe any methods used to assess certainty (or confidence) in the body of evidence for an outcome. | NA: No Meta |
| **RESULTS** | | |  |
| Study selection | 16a | Describe the results of the search and selection process, from the number of records identified in the search to the number of studies included in the review, ideally using a flow diagram. | Page 9; Fig 1 |
|  | 16b | Cite studies that might appear to meet the inclusion criteria, but which were excluded, and explain why they were excluded. | Fig 1 |
| Study characteristics | 17 | Cite each included study and present its characteristics. | Page 9-10; Table 1 |
| Risk of bias in studies | 18 | Present assessments of risk of bias for each included study. | Page 12; Table 1 |
| Results of individual studies | 19 | For all outcomes, present, for each study: (a) summary statistics for each group (where appropriate) and (b) an effect estimate and its precision (e.g. confidence/credible interval), ideally using structured tables or plots. | Page 13-20; Table 1-5 |
| Results of syntheses | 20a | For each synthesis, briefly summarise the characteristics and risk of bias among contributing studies. | Page 12; Table 1 |
|  | 20b | Present results of all statistical syntheses conducted. If meta-analysis was done, present for each the summary estimate and its precision (e.g. confidence/credible interval) and measures of statistical heterogeneity. If comparing groups, describe the direction of the effect. | NA: Narrative |
|  | 20c | Present results of all investigations of possible causes of heterogeneity among study results. | NA: Narrative |
|  | 20d | Present results of all sensitivity analyses conducted to assess the robustness of the synthesized results. | NA: Narrative |
| Reporting biases | 21 | Present assessments of risk of bias due to missing results (arising from reporting biases) for each synthesis assessed. | NA: Narrative |
| Certainty of evidence | 22 | Present assessments of certainty (or confidence) in the body of evidence for each outcome assessed. | NA: Narrative |
| **DISCUSSION** | | |  |
| Discussion | 23a | Provide a general interpretation of the results in the context of other evidence. | Page 20-23 |
|  | 23b | Discuss any limitations of the evidence included in the review. | Page 23-24 |
|  | 23c | Discuss any limitations of the review processes used. | Page 23-24 |
|  | 23d | Discuss implications of the results for practice, policy, and future research. | Page 24-25 |
| **OTHER INFORMATION** | | |  |
| Registration and protocol | 24a | Provide registration information for the review, including register name and registration number, or state that the review was not registered. | Page 6 |
|  | 24b | Indicate where the review protocol can be accessed, or state that a protocol was not prepared. | Page 6 |
|  | 24c | Describe and explain any amendments to information provided at registration or in the protocol. | Page 6 |
| Support | 25 | Describe sources of financial or non-financial support for the review, and the role of the funders or sponsors in the review. | Page 26 |
| Competing interests | 26 | Declare any competing interests of review authors. | Page 26 |
| Availability of data, code and other materials | 27 | Report which of the following are publicly available and where they can be found: template data collection forms; data extracted from included studies; data used for all analyses; analytic code; any other materials used in the review. | Page 26 |

S2. Full search strategies

BE-FIT Searches – March 9, 2021

**Medline – 3465**

| [# ▲](https://ovidsp-dc2-ovid-com.ezproxy.lib.ucalgary.ca/ovid-b/ovidweb.cgi?&S=DBEDFPJHNNEBILPPIPPJPEPEGOPMAA00&Sort+Sets=descending) | **Searches** | **Results** |
| --- | --- | --- |
| 1 | exp Environment Design/ | 7264 |
| 2 | (Walkab* or pedestrian* or "Land mix" or "land use mix" or traffic or road*).tw. | 92597 |
| 3 | ("population density" or "residential density" or "employment density" or "intersection density").tw. | 11143 |
| 4 | ("proximity to amenit* or proximity to service*" or "proximity to transit" or "proximity to park*").tw. | 42 |
| 5 | (road* or street*).tw. | 68223 |
| 6 | (destination* or "environmental modification*").tw. | 13426 |
| 7 | (footpath* or sidewalk* or "walking track*").tw. | 1263 |
| 8 | "cycl* lane*".tw. | 37 |
| 9 | "bike path*".tw. | 40 |
| 10 | ("cycl* path*" or "cycl track* or bicycl* path* or bicycl* track*").tw. | 3314 |
| 11 | ("green space*" or greenness or "public space*" or "public open space*" or park or parks or playground or "play* area*").tw. | 24943 |
| 12 | ("urban sprawl" or "car dependent").tw. | 429 |
| 13 | ("physical activity environment*" or "active living environment*" or "built environment*" or "healthy place*" or "urban environment" or "environment design" or "pedestrian environment" or "physical environment" or "urban form" or "urban design" or infrastructure or neighborhood* or neighbourhood* or "street lighting*" or "communit* design" or "Walk score").tw. | 79810 |
| 14 | ("Recreational facilit*" or "sports f*").tw. | 2231 |
| 15 | (1 or 2 or 3 or 4 or 5 or 6 or 7 or 8 or 9 or 10 or 11 or 12 or 13) and 14 | 355 |
| 16 | 1 or 2 or 3 or 4 or 5 or 6 or 7 or 8 or 9 or 10 or 11 or 12 or 13 or 15 | 230668 |
| 17 | ("aerobic* activit*" or "aerobic* fit*" or "aerobic* endur*" or "aerobic* capa*" or "aerobic* perform*" or "aerobic* test*" or "aerobic* str*").tw. | 10731 |
| 18 | ("cardio* activit*" or "cardio* fit*" or "cardio* endur*" or "cardio* capa*" or "cardio* perform*" or "cardio* test*" or "cardio* str*").tw. | 16378 |
| 19 | ("musc* activit*" or "musc* fit*" or "musc* endur*" or "musc* capa*" or "musc* perform*" or "musc* test*" or "musc* str*").tw. | 59775 |
| 20 | ((flexibility and health) or (flexibility and fitness) or (flexibility and physical) or (flexibility and activity) or "range of motion").tw. | 54591 |
| 21 | ((balance and health) or (balance and fitness) or (balance and physical) or (balance and activity)).tw. | 62883 |
| 22 | ("body comp*" or adip* lean*).tw. | 41343 |
| 23 | exp Body Composition/ | 56704 |
| 24 | ((health and fitness) or (physical and fitness) or (behavi* and fitness) or (functional and fitness)).tw. | 35001 |
| 25 | exp exercise test/ or exp physical fitness/ | 92522 |
| 26 | 17 or 18 or 19 or 20 or 21 or 22 or 23 or 24 or 25 | 356526 |
| 27 | 16 and 26 | 3465 |

**Embase – 4707**

| [# ▲](https://ovidsp-dc2-ovid-com.ezproxy.lib.ucalgary.ca/ovid-b/ovidweb.cgi?&S=DBEDFPJHNNEBILPPIPPJPEPEGOPMAA00&Sort+Sets=descending) | **Searches** | **Results** |
| --- | --- | --- |
| 1 | exp Environment Design/ | 14753 |
| 2 | (Walkab* or pedestrian* or "Land mix" or "land use mix" or traffic or road*).tw. | 115740 |
| 3 | ("population density" or "residential density" or "employment density" or "intersection density").tw. | 11508 |
| 4 | ("proximity to amenit* or proximity to service*" or "proximity to transit" or "proximity to park*").tw. | 47 |
| 5 | (road* or street*).tw. | 86015 |
| 6 | (destination* or "environmental modification*").tw. | 18065 |
| 7 | (footpath* or sidewalk* or "walking track*").tw. | 1518 |
| 8 | "cycl* lane*".tw. | 39 |
| 9 | "bike path*".tw. | 47 |
| 10 | ("cycl* path*" or "cycl track* or bicycl* path* or bicycl* track*").tw. | 4005 |
| 11 | ("green space*" or greenness or "public space*" or "public open space*" or park or parks or playground or "play* area*").tw. | 30741 |
| 12 | ("urban sprawl" or "car dependent").tw. | 487 |
| 13 | ("physical activity environment*" or "active living environment*" or "built environment*" or "healthy place*" or "urban environment" or "environment design" or "pedestrian environment" or "physical environment" or "urban form" or "urban design" or infrastructure or neighborhood* or neighbourhood* or "street lighting*" or "communit* design" or "Walk score").tw. | 95863 |
| 14 | ("Recreational facilit*" or "sports f*").tw. | 3050 |
| 15 | (1 or 2 or 3 or 4 or 5 or 6 or 7 or 8 or 9 or 10 or 11 or 12 or 13) and 14 | 410 |
| 16 | 1 or 2 or 3 or 4 or 5 or 6 or 7 or 8 or 9 or 10 or 11 or 12 or 13 or 15 | 290826 |
| 17 | ("aerobic* activit*" or "aerobic* fit*" or "aerobic* endur*" or "aerobic* capa*" or "aerobic* perform*" or "aerobic* test*" or "aerobic* str*").tw. | 13727 |
| 18 | ("cardio* activit*" or "cardio* fit*" or "cardio* endur*" or "cardio* capa*" or "cardio* perform*" or "cardio* test*" or "cardio* str*").tw. | 23120 |
| 19 | ("musc* activit*" or "musc* fit*" or "musc* endur*" or "musc* capa*" or "musc* perform*" or "musc* test*" or "musc* str*").tw. | 78310 |
| 20 | ((flexibility and health) or (flexibility and fitness) or (flexibility and physical) or (flexibility and activity) or "range of motion").tw. | 67678 |
| 21 | ((balance and health) or (balance and fitness) or (balance and physical) or (balance and activity)).tw. | 85075 |
| 22 | ("body comp*" or adip* lean*).tw. | 57296 |
| 23 | exp Body Composition/ | 106683 |
| 24 | ((health and fitness) or (physical and fitness) or (behavi* and fitness) or (functional and fitness)).tw. | 42731 |
| 25 | exp exercise test/ or exp physical fitness/ | 129306 |
| 26 | 17 or 18 or 19 or 20 or 21 or 22 or 23 or 24 or 25 | 484847 |
| 27 | 16 and 26 | 4707 |

**SPORTDiscus with full Text – 7346**

| **#** | **Query** | **Results** |
| --- | --- | --- |
| S28 | S16 AND S27 | 7,346 |
| S27 | S17 OR S18 OR S19 OR S20 OR S21 OR S22 OR S23 OR S24 OR S25 OR S26 | 208,640 |
| S26 | (TI (balance and health) or (balance and fitness) or (balance and physical) or (balance and activity) or (AB (balance and health) or (balance and fitness) or (balance and physical) or (balance and activity) or (KW (balance and health) or (balance and fitness) or (balance and physical) or (balance and activity) | 14,935 |
| S25 | (MH "Physical Fitness+") | 164 |
| S24 | (MH "Exercise Test+") | 197 |
| S23 | (TI (health and fitness) or (physical and fitness) or (behavi* and fitness) or (functional and fitness)) or (AB (health and fitness) or (physical and fitness) or (behavi* and fitness) or (functional and fitness)) or (KW (health and fitness) or (physical and fitness) or (behavi* and fitness) or (functional and fitness)) | 122,941 |
| S22 | (MH "Body Composition+") | 72 |
| S21 | (TI "body comp*" or adip* lean*) or (AB "body comp*" or adip* lean*) or (KW "body comp*" or adip* lean*) | 10,741 |
| S20 | (TI (flexibility and health) or (flexibility and fitness) or (flexibility and physical) or (flexibility and activity) or "range of motion") or (AB (flexibility and health) or (flexibility and fitness) or (flexibility and physical) or (flexibility and activity) or "range of motion") or (KW (flexibility and health) or (flexibility and fitness) or (flexibility and physical) or (flexibility and activity) or "range of motion") | 24,922 |
| S19 | (TI "musc* activit*" or "musc* fit*" or "musc* endur*" or "musc* capa*" or "musc* perform*" or "musc* test*" or "musc* str*") or (AB "musc* activit*" or "musc* fit*" or "musc* endur*" or "musc* capa*" or "musc* perform*" or "musc* test*" or "musc* str*") or (KW "musc* activit*" or "musc* fit*" or "musc* endur*" or "musc* capa*" or "musc* perform*" or "musc* test*" or "musc* str*") | 54,628 |
| S18 | (TI "cardio* activit*" or "cardio* fit*" or "cardio* endur*" or "cardio* capa*" or "cardio* perform*" or "cardio* test*" or "cardio* str*") or (AB "cardio* activit*" or "cardio* fit*" or "cardio* endur*" or "cardio* capa*" or "cardio* perform*" or "cardio* test*" or "cardio* str*") or (KW "cardio* activit*" or "cardio* fit*" or "cardio* endur*" or "cardio* capa*" or "cardio* perform*" or "cardio* test*" or "cardio* str*") | 5,988 |
| S17 | (TI "aerobic* activit*" or "aerobic* fit*" or "aerobic* endur*" or "aerobic* capa*" or "aerobic* perform*" or "aerobic* test*" or "aerobic* str*") or (AB "aerobic* activit*" or "aerobic* fit*" or "aerobic* endur*" or "aerobic* capa*" or "aerobic* perform*" or "aerobic* test*" or "aerobic* str*") or (KW "aerobic* activit*" or "aerobic* fit*" or "aerobic* endur*" or "aerobic* capa*" or "aerobic* perform*" or "aerobic* test*" or "aerobic* str*") | 14,800 |
| S16 | S14 OR S15 | 162,332 |
| S15 | S13 AND S14 | 46,702 |
| S14 | S1 OR S2 OR S3 OR S4 OR S5 OR S6 OR S7 OR S8 OR S9 OR S10 OR S11 OR S12 OR S12 | 162,332 |
| S13 | (TI "Recreational facilit*" or "sports f*") or (AB "Recreational facilit*" or "sports f*") or (KW "Recreational facilit*" or "sports f*") | 718,460 |
| S12 | (TI "physical activity environment*" or "active living environment*" or "built environment*" or "healthy place*" or "urban environment" or "environment design" or "pedestrian environment" or "physical environment" or "urban form" or "urban design" or infrastructure or neighborhood* or neighbourhood* or "street lighting*" or "communit* design" or "Walk score") or (AB "physical activity environment*" or "active living environment*" or "built environment*" or "healthy place*" or "urban environment" or "environment design" or "pedestrian environment" or "physical environment" or "urban form" or "urban design" or infrastructure or neighborhood* or neighbourhood* or "street lighting*" or "communit* design" or "Walk score") or (KW "physical activity environment*" or "active living environment*" or "built environment*" or "healthy place*" or "urban environment" or "environment design" or "pedestrian environment" or "physical environment" or "urban form" or "urban design" or infrastructure or neighborhood* or neighbourhood* or "street lighting*" or "communit* design" or "Walk score") | 7,565 |
| S11 | (TI "urban sprawl" or "car dependent") or (AB "urban sprawl" or "car dependent") or (KW "urban sprawl" or "car dependent") | 38 |
| S10 | (TI "green space*" or greenness or "public space*" or "public open space*" or park or parks or playground or "play* area*") or (AB "green space*" or greenness or "public space*" or "public open space*" or park or parks or playground or "play* area*") or (KW "green space*" or greenness or "public space*" or "public open space*" or park or parks or playground or "play* area*") | 63,832 |
| S9 | (TI "cycl* path*" or "cycl track*" or "bicycl* path*" or "bicycl* track*") or (AB "cycl* path*" or "cycl track*" or "bicycl* path* or "bicycl* track*") or (KW "cycl* path*" or "cycl track*" or "bicycl* path* or "bicycl* track*") | 151 |
| S8 | (TI "bike path*") or (AB "bike path*") or (KW "bike path*") | 83 |
| S7 | (TI "cycl* lane*") or (AB "cycl* lane*") or (KW "cycl* lane*") | 43 |
| S6 | (TI footpath* or sidewalk* or "walking track*") or (AB footpath* or sidewalk* or "walking track*") or (KW footpath* or sidewalk* or "walking track*") | 362 |
| S5 | (TI destination* or "environmental modification*") or (AB destination* or "environmental modification*") or (KW destination* or "environmental modification*") | 6,846 |
| S4 | (TI road* or street*) or (AB road* or street*) or (KW road* or street*) | 71,364 |
| S3 | (TI "proximity to amenit* or proximity to service*" or "proximity to transit" or "proximity to park*") or (AB "proximity to amenit* or proximity to service*" or "proximity to transit" or "proximity to park*") or (KW "proximity to amenit* or proximity to service*" or "proximity to transit" or "proximity to park*") | 9 |
| S2 | (TI "population density" or "residential density" or "employment density" or "intersection density") or (AB "population density" or "residential density" or "employment density" or "intersection density") or (KW "population density" or "residential density" or "employment density" or "intersection density") | 263 |
| S1 | (TI Walkab* or pedestrian* or "Land mix" or "land use mix" or traffic or road*) or (AB Walkab* or pedestrian* or "Land mix" or "land use mix" or traffic or road*) or (TW Walkab* or pedestrian* or "Land mix" or "land use mix" or traffic or road*) | 45,610 |

**Environment Complete - 6060**

| **#** | **Query** | **Results** |
| --- | --- | --- |
| S28 | S16 AND S27 | 6,060 |
| S27 | S17 OR S18 OR S19 OR S20 OR S21 OR S22 OR S23 OR S24 OR S25 OR S26 | 44,468 |
| S26 | (TI (balance and health) or (balance and fitness) or (balance and physical) or (balance and activity) or (AB (balance and health) or (balance and fitness) or (balance and physical) or (balance and activity) or (KW (balance and health) or (balance and fitness) or (balance and physical) or (balance and activity) | 19,090 |
| S25 | (MH "Physical Fitness+") | 5,487 |
| S24 | (MH "Exercise Test+") | 183 |
| S23 | (TI (health and fitness) or (physical and fitness) or (behavi* and fitness) or (functional and fitness)) or (AB (health and fitness) or (physical and fitness) or (behavi* and fitness) or (functional and fitness)) or (KW (health and fitness) or (physical and fitness) or (behavi* and fitness) or (functional and fitness)) | 14,392 |
| S22 | (MH "Body Composition+") | 227 |
| S21 | (TI "body comp*" or adip* lean*) or (AB "body comp*" or adip* lean*) or (KW "body comp*" or adip* lean*) | 5,348 |
| S20 | (TI (flexibility and health) or (flexibility and fitness) or (flexibility and physical) or (flexibility and activity) or "range of motion") or (AB (flexibility and health) or (flexibility and fitness) or (flexibility and physical) or (flexibility and activity) or "range of motion") or (KW (flexibility and health) or (flexibility and fitness) or (flexibility and physical) or (flexibility and activity) or "range of motion") | 4,353 |
| S19 | (TI "musc* activit*" or "musc* fit*" or "musc* endur*" or "musc* capa*" or "musc* perform*" or "musc* test*" or "musc* str*") or (AB "musc* activit*" or "musc* fit*" or "musc* endur*" or "musc* capa*" or "musc* perform*" or "musc* test*" or "musc* str*") or (KW "musc* activit*" or "musc* fit*" or "musc* endur*" or "musc* capa*" or "musc* perform*" or "musc* test*" or "musc* str*") | 2,004 |
| S18 | (TI "cardio* activit*" or "cardio* fit*" or "cardio* endur*" or "cardio* capa*" or "cardio* perform*" or "cardio* test*" or "cardio* str*") or (AB "cardio* activit*" or "cardio* fit*" or "cardio* endur*" or "cardio* capa*" or "cardio* perform*" or "cardio* test*" or "cardio* str*") or (KW "cardio* activit*" or "cardio* fit*" or "cardio* endur*" or "cardio* capa*" or "cardio* perform*" or "cardio* test*" or "cardio* str*") | 570 |
| S17 | (TI "aerobic* activit*" or "aerobic* fit*" or "aerobic* endur*" or "aerobic* capa*" or "aerobic* perform*" or "aerobic* test*" or "aerobic* str*") or (AB "aerobic* activit*" or "aerobic* fit*" or "aerobic* endur*" or "aerobic* capa*" or "aerobic* perform*" or "aerobic* test*" or "aerobic* str*") or (KW "aerobic* activit*" or "aerobic* fit*" or "aerobic* endur*" or "aerobic* capa*" or "aerobic* perform*" or "aerobic* test*" or "aerobic* str*") | 812 |
| S16 | S14 OR S15 | 543,124 |
| S15 | S13 AND S14 | 4,269 |
| S14 | S1 OR S2 OR S3 OR S4 OR S5 OR S6 OR S7 OR S8 OR S9 OR S10 OR S11 OR S12 OR S12 | 543,124 |
| S13 | (TI "Recreational facilit*" or "sports f*") or (AB "Recreational facilit*" or "sports f*") or (KW "Recreational facilit*" or "sports f*") | 19,377 |
| S12 | (TI "physical activity environment*" or "active living environment*" or "built environment*" or "healthy place*" or "urban environment" or "environment design" or "pedestrian environment" or "physical environment" or "urban form" or "urban design" or infrastructure or neighborhood* or neighbourhood* or "street lighting*" or "communit* design" or "Walk score") or (AB "physical activity environment*" or "active living environment*" or "built environment*" or "healthy place*" or "urban environment" or "environment design" or "pedestrian environment" or "physical environment" or "urban form" or "urban design" or infrastructure or neighborhood* or neighbourhood* or "street lighting*" or "communit* design" or "Walk score") or (KW "physical activity environment*" or "active living environment*" or "built environment*" or "healthy place*" or "urban environment" or "environment design" or "pedestrian environment" or "physical environment" or "urban form" or "urban design" or infrastructure or neighborhood* or neighbourhood* or "street lighting*" or "communit* design" or "Walk score") | 88,749 |
| S11 | (TI "urban sprawl" or "car dependent") or (AB "urban sprawl" or "car dependent") or (KW "urban sprawl" or "car dependent") | 1,480 |
| S10 | (TI "green space*" or greenness or "public space*" or "public open space*" or park or parks or playground or "play* area*") or (AB "green space*" or greenness or "public space*" or "public open space*" or park or parks or playground or "play* area*") or (KW "green space*" or greenness or "public space*" or "public open space*" or park or parks or playground or "play* area*") | 179,637 |
| S9 | (TI "cycl* path*" or "cycl track*" or "bicycl* path*" or "bicycl* track*") or (AB "cycl* path*" or "cycl track*" or "bicycl* path* or "bicycl* track*") or (KW "cycl* path*" or "cycl track*" or "bicycl* path* or "bicycl* track*") | 523 |
| S8 | (TI "bike path*") or (AB "bike path*") or (KW "bike path*") | 53 |
| S7 | (TI "cycl* lane*") or (AB "cycl* lane*") or (KW "cycl* lane*") | 26 |
| S6 | (TI footpath* or sidewalk* or "walking track*") or (AB footpath* or sidewalk* or "walking track*") or (KW footpath* or sidewalk* or "walking track*") | 1,628 |
| S5 | (TI destination* or "environmental modification*") or (AB destination* or "environmental modification*") or (KW destination* or "environmental modification*") | 5,863 |
| S4 | (TI road* or street*) or (AB road* or street*) or (KW road* or street*) | 152,466 |
| S3 | (TI "proximity to amenit* or proximity to service*" or "proximity to transit" or "proximity to park*") or (AB "proximity to amenit* or proximity to service*" or "proximity to transit" or "proximity to park*") or (KW "proximity to amenit* or proximity to service*" or "proximity to transit" or "proximity to park*") | 33 |
| S2 | (TI "population density" or "residential density" or "employment density" or "intersection density") or (AB "population density" or "residential density" or "employment density" or "intersection density") or (KW "population density" or "residential density" or "employment density" or "intersection density") | 12,101 |
| S1 | (TI Walkab* or pedestrian* or "Land mix" or "land use mix" or traffic or road*) or (AB Walkab* or pedestrian* or "Land mix" or "land use mix" or traffic or road*) or (TW Walkab* or pedestrian* or "Land mix" or "land use mix" or traffic or road*) | 224,997 |

**CINAHL Plus with Full Text - 2834**

| **#** | **Query** | **Results** |
| --- | --- | --- |
| S28 | S16 AND S27 | 2,834 |
| S27 | S17 OR S18 OR S19 OR S20 OR S21 OR S22 OR S23 OR S24 OR S25 OR S26 | 139,466 |
| S26 | (TI (balance and health) or (balance and fitness) or (balance and physical) or (balance and activity) or (AB (balance and health) or (balance and fitness) or (balance and physical) or (balance and activity) or (KW (balance and health) or (balance and fitness) or (balance and physical) or (balance and activity) | 24,585 |
| S25 | (MH "Physical Fitness+") | 1,000 |
| S24 | (MH "Exercise Test+") | 2,758 |
| S23 | (TI (health and fitness) or (physical and fitness) or (behavi* and fitness) or (functional and fitness)) or (AB (health and fitness) or (physical and fitness) or (behavi* and fitness) or (functional and fitness)) or (KW (health and fitness) or (physical and fitness) or (behavi* and fitness) or (functional and fitness)) | 27,695 |
| S22 | (MH "Body Composition+") | 1,064 |
| S21 | (TI "body comp*" or adip* lean*) or (AB "body comp*" or adip* lean*) or (KW "body comp*" or adip* lean*) | 15,281 |
| S20 | (TI (flexibility and health) or (flexibility and fitness) or (flexibility and physical) or (flexibility and activity) or "range of motion") or (AB (flexibility and health) or (flexibility and fitness) or (flexibility and physical) or (flexibility and activity) or "range of motion") or (KW (flexibility and health) or (flexibility and fitness) or (flexibility and physical) or (flexibility and activity) or "range of motion") | 41,205 |
| S19 | (TI "musc* activit*" or "musc* fit*" or "musc* endur*" or "musc* capa*" or "musc* perform*" or "musc* test*" or "musc* str*") or (AB "musc* activit*" or "musc* fit*" or "musc* endur*" or "musc* capa*" or "musc* perform*" or "musc* test*" or "musc* str*") or (KW "musc* activit*" or "musc* fit*" or "musc* endur*" or "musc* capa*" or "musc* perform*" or "musc* test*" or "musc* str*") | 43,123 |
| S18 | (TI "cardio* activit*" or "cardio* fit*" or "cardio* endur*" or "cardio* capa*" or "cardio* perform*" or "cardio* test*" or "cardio* str*") or (AB "cardio* activit*" or "cardio* fit*" or "cardio* endur*" or "cardio* capa*" or "cardio* perform*" or "cardio* test*" or "cardio* str*") or (KW "cardio* activit*" or "cardio* fit*" or "cardio* endur*" or "cardio* capa*" or "cardio* perform*" or "cardio* test*" or "cardio* str*") | 6,376 |
| S17 | (TI "aerobic* activit*" or "aerobic* fit*" or "aerobic* endur*" or "aerobic* capa*" or "aerobic* perform*" or "aerobic* test*" or "aerobic* str*") or (AB "aerobic* activit*" or "aerobic* fit*" or "aerobic* endur*" or "aerobic* capa*" or "aerobic* perform*" or "aerobic* test*" or "aerobic* str*") or (KW "aerobic* activit*" or "aerobic* fit*" or "aerobic* endur*" or "aerobic* capa*" or "aerobic* perform*" or "aerobic* test*" or "aerobic* str*") | 5,675 |
| S16 | S14 OR S15 | 131,291 |
| S15 | S13 AND S14 | 1,541 |
| S14 | S1 OR S2 OR S3 OR S4 OR S5 OR S6 OR S7 OR S8 OR S9 OR S10 OR S11 OR S12 OR S12 | 131,291 |
| S13 | (TI "Recreational facilit*" or "sports f*") or (AB "Recreational facilit*" or "sports f*") or (KW "Recreational facilit*" or "sports f*") | 59,974 |
| S12 | (TI "physical activity environment*" or "active living environment*" or "built environment*" or "healthy place*" or "urban environment" or "environment design" or "pedestrian environment" or "physical environment" or "urban form" or "urban design" or infrastructure or neighborhood* or neighbourhood* or "street lighting*" or "communit* design" or "Walk score") or (AB "physical activity environment*" or "active living environment*" or "built environment*" or "healthy place*" or "urban environment" or "environment design" or "pedestrian environment" or "physical environment" or "urban form" or "urban design" or infrastructure or neighborhood* or neighbourhood* or "street lighting*" or "communit* design" or "Walk score") or (KW "physical activity environment*" or "active living environment*" or "built environment*" or "healthy place*" or "urban environment" or "environment design" or "pedestrian environment" or "physical environment" or "urban form" or "urban design" or infrastructure or neighborhood* or neighbourhood* or "street lighting*" or "communit* design" or "Walk score") | 31,908 |
| S11 | (TI "urban sprawl" or "car dependent") or (AB "urban sprawl" or "car dependent") or (KW "urban sprawl" or "car dependent") | 69 |
| S10 | (TI "green space*" or greenness or "public space*" or "public open space*" or park or parks or playground or "play* area*") or (AB "green space*" or greenness or "public space*" or "public open space*" or park or parks or playground or "play* area*") or (KW "green space*" or greenness or "public space*" or "public open space*" or park or parks or playground or "play* area*") | 48,054 |
| S9 | (TI "cycl* path*" or "cycl track*" or "bicycl* path*" or "bicycl* track*") or (AB "cycl* path*" or "cycl track*" or "bicycl* path* or "bicycl* track*") or (KW "cycl* path*" or "cycl track*" or "bicycl* path* or "bicycl* track*") | 206 |
| S8 | (TI "bike path*") or (AB "bike path*") or (KW "bike path*") | 28 |
| S7 | (TI "cycl* lane*") or (AB "cycl* lane*") or (KW "cycl* lane*") | 8 |
| S6 | (TI footpath* or sidewalk* or "walking track*") or (AB footpath* or sidewalk* or "walking track*") or (KW footpath* or sidewalk* or "walking track*") | 430 |
| S5 | (TI destination* or "environmental modification*") or (AB destination* or "environmental modification*") or (KW destination* or "environmental modification*") | 4,307 |
| S4 | (TI road* or street*) or (AB road* or street*) or (KW road* or street*) | 30,953 |
| S3 | (TI "proximity to amenit* or proximity to service*" or "proximity to transit" or "proximity to park*") or (AB "proximity to amenit* or proximity to service*" or "proximity to transit" or "proximity to park*") or (KW "proximity to amenit* or proximity to service*" or "proximity to transit" or "proximity to park*") | 22 |
| S2 | (TI "population density" or "residential density" or "employment density" or "intersection density") or (AB "population density" or "residential density" or "employment density" or "intersection density") or (KW "population density" or "residential density" or "employment density" or "intersection density") | 1,287 |
| S1 | (TI Walkab* or pedestrian* or "Land mix" or "land use mix" or traffic or road*) or (AB Walkab* or pedestrian* or "Land mix" or "land use mix" or traffic or road*) or (TW Walkab* or pedestrian* or "Land mix" or "land use mix" or traffic or road*) | 37,102 |

**Web of Science – 11440**

| # 25 | [**11,440**](https://apps-webofknowledge-com.ezproxy.lib.ucalgary.ca/summary.do?product=WOS&doc=1&qid=25&SID=5ANeMMlcXrcguhRGHva&search_mode=CombineSearches&update_back2search_link_param=yes) | #24  AND  #16  *Indexes=SCI-EXPANDED, SSCI, A&HCI, CPCI-S, CPCI-SSH, ESCI Timespan=All years* |
| --- | --- | --- |
| # 24 | [**439,337**](https://apps-webofknowledge-com.ezproxy.lib.ucalgary.ca/summary.do?product=WOS&doc=1&qid=24&SID=5ANeMMlcXrcguhRGHva&search_mode=CombineSearches&update_back2search_link_param=yes) | #23  OR  #22  OR  #21  OR  #20  OR  #19  OR  #18  OR  #17  *Indexes=SCI-EXPANDED, SSCI, A&HCI, CPCI-S, CPCI-SSH, ESCI Timespan=All years* |
| # 23 | [**151,473**](https://apps-webofknowledge-com.ezproxy.lib.ucalgary.ca/summary.do?product=WOS&doc=1&qid=23&SID=5ANeMMlcXrcguhRGHva&search_mode=AdvancedSearch&update_back2search_link_param=yes) | TS=((balance  and  health)  or  (balance and fitness)  or  (balance and physical)  or  (balance and activity)  )  *Indexes=SCI-EXPANDED, SSCI, A&HCI, CPCI-S, CPCI-SSH, ESCI Timespan=All years* |
| # 22 | [**70,576**](https://apps-webofknowledge-com.ezproxy.lib.ucalgary.ca/summary.do?product=WOS&doc=1&qid=22&SID=5ANeMMlcXrcguhRGHva&search_mode=AdvancedSearch&update_back2search_link_param=yes) | TS=((health  and  fitness)  or  (physical and fitness)  or  (behavi* and fitness)  or  (functional and fitness)  )  *Indexes=SCI-EXPANDED, SSCI, A&HCI, CPCI-S, CPCI-SSH, ESCI Timespan=All years* |
| # 21 | [**82,610**](https://apps-webofknowledge-com.ezproxy.lib.ucalgary.ca/summary.do?product=WOS&doc=1&qid=21&SID=5ANeMMlcXrcguhRGHva&search_mode=AdvancedSearch&update_back2search_link_param=yes) | TS=("body  comp*"  or  adip*  lean*)  *Indexes=SCI-EXPANDED, SSCI, A&HCI, CPCI-S, CPCI-SSH, ESCI Timespan=All years* |
| # 20 | [**74,166**](https://apps-webofknowledge-com.ezproxy.lib.ucalgary.ca/summary.do?product=WOS&doc=1&qid=20&SID=5ANeMMlcXrcguhRGHva&search_mode=AdvancedSearch&update_back2search_link_param=yes) | TS=((flexibility  and  health)  or  (flexibility and fitness)  or  (flexibility and physical)  or  (flexibility and activity)  or  "range  of  motion")  *Indexes=SCI-EXPANDED, SSCI, A&HCI, CPCI-S, CPCI-SSH, ESCI Timespan=All years* |
| # 19 | [**72,871**](https://apps-webofknowledge-com.ezproxy.lib.ucalgary.ca/summary.do?product=WOS&doc=1&qid=19&SID=5ANeMMlcXrcguhRGHva&search_mode=AdvancedSearch&update_back2search_link_param=yes) | TS=("musc*  activit*"  or  "musc*  fit*"  or  "musc*  endur*"  or  "musc*  capa*"  or  "musc*  perform*"  or  "musc*  test*"  or  "musc*  str*")  *Indexes=SCI-EXPANDED, SSCI, A&HCI, CPCI-S, CPCI-SSH, ESCI Timespan=All years* |
| # 18 | [**22,037**](https://apps-webofknowledge-com.ezproxy.lib.ucalgary.ca/summary.do?product=WOS&doc=1&qid=18&SID=5ANeMMlcXrcguhRGHva&search_mode=AdvancedSearch&update_back2search_link_param=yes) | TS=("cardio*  activit*"  or  "cardio*  fit*"  or  "cardio*  endur*"  or  "cardio*  capa*"  or  "cardio*  perform*"  or  "cardio*  test*"  or  "cardio*  str*")  *Indexes=SCI-EXPANDED, SSCI, A&HCI, CPCI-S, CPCI-SSH, ESCI Timespan=All years* |
| # 17 | [**15,457**](https://apps-webofknowledge-com.ezproxy.lib.ucalgary.ca/summary.do?product=WOS&doc=1&qid=17&SID=5ANeMMlcXrcguhRGHva&search_mode=AdvancedSearch&update_back2search_link_param=yes) | TS=("aerobic*  activit*"  or  "aerobic*  fit*"  or  "aerobic*  endur*"  or  "aerobic*  capa*"  or  "aerobic*  perform*"  or  "aerobic*  test*"  or  "aerobic*  str*")  *Indexes=SCI-EXPANDED, SSCI, A&HCI, CPCI-S, CPCI-SSH, ESCI Timespan=All years* |
| # 16 | [**1,232,512**](https://apps-webofknowledge-com.ezproxy.lib.ucalgary.ca/summary.do?product=WOS&doc=1&qid=16&SID=5ANeMMlcXrcguhRGHva&search_mode=CombineSearches&update_back2search_link_param=yes) | #15 OR #14  *Indexes=SCI-EXPANDED, SSCI, A&HCI, CPCI-S, CPCI-SSH, ESCI Timespan=All years* |
| # 15 | [**931**](https://apps-webofknowledge-com.ezproxy.lib.ucalgary.ca/summary.do?product=WOS&doc=1&qid=15&SID=5ANeMMlcXrcguhRGHva&search_mode=CombineSearches&update_back2search_link_param=yes) | #14  AND  #13  *Indexes=SCI-EXPANDED, SSCI, A&HCI, CPCI-S, CPCI-SSH, ESCI Timespan=All years* |
| # 14 | [**1,232,512**](https://apps-webofknowledge-com.ezproxy.lib.ucalgary.ca/summary.do?product=WOS&doc=1&qid=14&SID=5ANeMMlcXrcguhRGHva&search_mode=AdvancedSearch&update_back2search_link_param=yes) | #12  OR  #11  OR  #10  OR  #9  OR  #8  OR  #7  OR  #6  OR  #5  OR  #4  OR  #3  OR  #2  OR  #1  *Indexes=SCI-EXPANDED, SSCI, A&HCI, CPCI-S, CPCI-SSH, ESCI Timespan=All years* |
| # 13 | [**5,635**](https://apps-webofknowledge-com.ezproxy.lib.ucalgary.ca/summary.do?product=WOS&doc=1&qid=13&SID=5ANeMMlcXrcguhRGHva&search_mode=AdvancedSearch&update_back2search_link_param=yes) | TS=("Recreational  facilit*"  or  "sports  f*")  *Indexes=SCI-EXPANDED, SSCI, A&HCI, CPCI-S, CPCI-SSH, ESCI Timespan=All years* |
| # 12 | [**420,183**](https://apps-webofknowledge-com.ezproxy.lib.ucalgary.ca/summary.do?product=WOS&doc=1&qid=12&SID=5ANeMMlcXrcguhRGHva&search_mode=AdvancedSearch&update_back2search_link_param=yes) | TS=("physical  activity  environment*"  or  "active  living  environment*"  or  "built  environment*"  or  "healthy  place*"  or  "urban  environment"  or  "environment  design"  or  "pedestrian  environment"  or  "physical  environment"  or  "urban  form"  or  "urban  design"  or  infrastructure  or  neighborhood*  or  neighbourhood*  or  "street  lighting*"  or  "communit*  design"  or  "Walk  score")  *Indexes=SCI-EXPANDED, SSCI, A&HCI, CPCI-S, CPCI-SSH, ESCI Timespan=All years* |
| # 11 | [**4,207**](https://apps-webofknowledge-com.ezproxy.lib.ucalgary.ca/summary.do?product=WOS&doc=1&qid=11&SID=5ANeMMlcXrcguhRGHva&search_mode=AdvancedSearch&update_back2search_link_param=yes) | TS=("urban  sprawl"  or  "car  dependent")  *Indexes=SCI-EXPANDED, SSCI, A&HCI, CPCI-S, CPCI-SSH, ESCI Timespan=All years* |
| # 10 | [**145,446**](https://apps-webofknowledge-com.ezproxy.lib.ucalgary.ca/summary.do?product=WOS&doc=1&qid=10&SID=5ANeMMlcXrcguhRGHva&search_mode=AdvancedSearch&update_back2search_link_param=yes) | TS=("green  space*"  or  greenness  or  "public  space*"  or  "public  open  space*"  or  park  or  parks  or  playground  or  "play*  area*")  *Indexes=SCI-EXPANDED, SSCI, A&HCI, CPCI-S, CPCI-SSH, ESCI Timespan=All years* |
| # 9 | [**4,108**](https://apps-webofknowledge-com.ezproxy.lib.ucalgary.ca/summary.do?product=WOS&doc=1&qid=9&SID=5ANeMMlcXrcguhRGHva&search_mode=AdvancedSearch&update_back2search_link_param=yes) | TS=("cycl*  path*"  or  "cycl  track*"  or  "bicycl*  path*"  or  "bicycl*  track*")  *Indexes=SCI-EXPANDED, SSCI, A&HCI, CPCI-S, CPCI-SSH, ESCI Timespan=All years* |
| # 8 | [**136**](https://apps-webofknowledge-com.ezproxy.lib.ucalgary.ca/summary.do?product=WOS&doc=1&qid=8&SID=5ANeMMlcXrcguhRGHva&search_mode=AdvancedSearch&update_back2search_link_param=yes) | TS=("bike  path*")  *Indexes=SCI-EXPANDED, SSCI, A&HCI, CPCI-S, CPCI-SSH, ESCI Timespan=All years* |
| # 7 | [**112**](https://apps-webofknowledge-com.ezproxy.lib.ucalgary.ca/summary.do?product=WOS&doc=1&qid=7&SID=5ANeMMlcXrcguhRGHva&search_mode=AdvancedSearch&update_back2search_link_param=yes) | TS=("cycl*  lane*")  *Indexes=SCI-EXPANDED, SSCI, A&HCI, CPCI-S, CPCI-SSH, ESCI Timespan=All years* |
| # 6 | [**3,456**](https://apps-webofknowledge-com.ezproxy.lib.ucalgary.ca/summary.do?product=WOS&doc=1&qid=6&SID=5ANeMMlcXrcguhRGHva&search_mode=AdvancedSearch&update_back2search_link_param=yes) | TS=(footpath*  or  sidewalk*  or  "walking  track*")  *Indexes=SCI-EXPANDED, SSCI, A&HCI, CPCI-S, CPCI-SSH, ESCI Timespan=All years* |
| # 5 | [**79,927**](https://apps-webofknowledge-com.ezproxy.lib.ucalgary.ca/summary.do?product=WOS&doc=1&qid=5&SID=5ANeMMlcXrcguhRGHva&search_mode=AdvancedSearch&update_back2search_link_param=yes) | TS=(destination*  or  "environmental  modification*")  *Indexes=SCI-EXPANDED, SSCI, A&HCI, CPCI-S, CPCI-SSH, ESCI Timespan=All years* |
| # 4 | [**313,502**](https://apps-webofknowledge-com.ezproxy.lib.ucalgary.ca/summary.do?product=WOS&doc=1&qid=4&SID=5ANeMMlcXrcguhRGHva&search_mode=AdvancedSearch&update_back2search_link_param=yes) | TS=(road*  or  street*)  *Indexes=SCI-EXPANDED, SSCI, A&HCI, CPCI-S, CPCI-SSH, ESCI Timespan=All years* |
| # 3 | [**138**](https://apps-webofknowledge-com.ezproxy.lib.ucalgary.ca/summary.do?product=WOS&doc=1&qid=3&SID=5ANeMMlcXrcguhRGHva&search_mode=AdvancedSearch&update_back2search_link_param=yes) | TS=("proximity  to  amenit*"  or  "proximity  to  service*"  or  "proximity  to  transit"  or  "proximity  to  park*")  *Indexes=SCI-EXPANDED, SSCI, A&HCI, CPCI-S, CPCI-SSH, ESCI Timespan=All years* |
| # 2 | [**32,311**](https://apps-webofknowledge-com.ezproxy.lib.ucalgary.ca/summary.do?product=WOS&doc=1&qid=2&SID=5ANeMMlcXrcguhRGHva&search_mode=AdvancedSearch&update_back2search_link_param=yes) | TS=("population  density"  or  "residential  density"  or  "employment  density"  or  "intersection  density")  *Indexes=SCI-EXPANDED, SSCI, A&HCI, CPCI-S, CPCI-SSH, ESCI Timespan=All years* |
| # 1 | [**582,957**](https://apps-webofknowledge-com.ezproxy.lib.ucalgary.ca/summary.do?product=WOS&doc=1&qid=1&SID=5ANeMMlcXrcguhRGHva&search_mode=AdvancedSearch&update_back2search_link_param=yes) | TS=(Walkab*  or  pedestrian*  or  "Land  mix"  or  "land  use  mix"  or  traffic  or  road*)  *Indexes=SCI-EXPANDED, SSCI, A&HCI, CPCI-S, CPCI-SSH, ESCI Timespan=All years* |

**ProQuest Dissertations & Theses Global – 3234**

**(noft(Walkab* OR pedestrian* OR "Land mix" OR "land use mix" OR traffic OR road*) OR noft("population density" OR "residential density" OR "employment density" OR "intersection density") OR noft(road* OR street*) OR noft(destination* OR "environmental modification*") OR noft(footpath* OR sidewalk* OR "walking track*") OR noft("cycl* lane*") OR noft("bike path*") OR noft("cycl* path*" OR "cycl track*" OR "bicycl* path*" OR "bicycl* track*") OR noft("green space*" OR greenness OR "public space*" OR "public open space*" OR park OR parks OR playground OR "play* area*") OR noft("urban sprawl" OR "car dependent") OR noft("proximity to amenit* or proximity to service*" OR "proximity to transit" OR "proximity to park*") OR noft("physical activity environment*" OR "active living environment*" OR "built environment*" OR "healthy place*" OR "urban environment" OR "environment design" OR "pedestrian environment" OR "physical environment" OR "urban form" OR "urban design" OR infrastructure OR neighborhood* OR neighbourhood* OR "street lighting*" OR "communit* design" OR "Walk score")) AND (noft("aerobic* activit*" OR "aerobic* fit*" OR "aerobic* endur*" OR "aerobic* capa*" OR "aerobic* perform*" OR "aerobic* test*" OR "aerobic* str*") OR noft("cardio* activit*" OR "cardio* fit*" OR "cardio* endur*" OR "cardio* capa*" OR "cardio* perform*" OR "cardio* test*" OR "cardio* str*") OR noft("musc* activit*" OR "musc* fit*" OR "musc* endur*" OR "musc* capa*" OR "musc* perform*" OR "musc* test*" OR "musc* str*") OR noft((flexibility AND health) OR (flexibility AND fitness) OR (flexibility AND physical) OR (flexibility AND activity) OR "range of motion") OR noft("body comp*" OR adip* lean*) OR noft((health AND fitness) OR (physical AND fitness) OR (behavi* AND fitness) OR (functional AND fitness)) OR noft((balance AND health) OR (balance AND fitness) OR (balance AND physical) OR (balance AND activity)))**

**TRID – 490**

#25

490

24 and 16

#24

3953

23 or 22 or 21 or 20 or 19 or 18 or 17

#23

868

You searched with keywords containing **((balance and health) or (balance and fitness) or (balance and physical) or (balance and activity) )**

#22

1672

You searched with keywords containing **((health and fitness) or (physical and fitness) or (behavi* and fitness) or (functional and fitness) )**

#21

448

You searched with keywords containing **("body comp*" or adip* lean*)**

#20

884

You searched with keywords containing **((flexibility and health) or (flexibility and fitness) or (flexibility and physical) or (flexibility and activity) or "range of motion")**

#19

162

You searched with keywords containing **("musc* activit*" or "musc* fit*" or "musc* endur*" or "musc* capa*" or "musc* perform*" or "musc* test*" or "musc* str*")**

#18

27

You searched with keywords containing **("cardio* activit*" or "cardio* fit*" or "cardio* endur*" or "cardio* capa*" or "cardio* perform*" or "cardio* test*" or "cardio* str*")**

#17

3

You searched with keywords containing **("aerobic* activit*" or "aerobic* fit*" or "aerobic* endur*" or "aerobic* capa*" or "aerobic* perform*" or "aerobic* test*" or "aerobic* str*")**

#16

69187

15 or 14

#15

189

14 and 13

#14

69187

12 or 11 or 10 or 9 or 8 or 7 or 6 or 5 or 4 or 3 or 2 or 1

#13

682

You searched with keywords containing **("Recreational facilit*" or "sports f*")**

#12

15000

You searched with keywords containing **("physical activity environment*" or "active living environment*" or "built environment*" or "healthy place*" or "urban environment" or "environment design" or "pedestrian environment" or "physical environment" or "urban form" or "urban design" or infrastructure or neighborhood* or neighbourhood* or "street lighting*" or "communit* design" or "Walk score")**

#11

1137

You searched with keywords containing **("urban sprawl" or "car dependent")**

#10

15000

You searched with keywords containing **("green space*" or greenness or "public space*" or "public open space*" or park or parks or playground or "play* area*")**

#9

3877

You searched with keywords containing **("cycl* path*" or "cycl track*" or "bicycl* path*" or "bicycl* track*")**

#8

213

You searched with keywords containing **("bike path*")**

#7

912

You searched with keywords containing **("cycl* lane*")**

#6

3161

You searched with keywords containing **(footpath* or sidewalk* or "walking track*")**

#5

15000

You searched with keywords containing **(destination* or "environmental modification*")**

#4

15000

You searched with keywords containing **(road* or street*)**

#3

61

You searched with keywords containing **("proximity to amenit*" or "proximity to service*" or "proximity to transit" or "proximity to park*")**

#2

2512

You searched with keywords containing **("population density" or "residential density" or "employment density" or "intersection density")**

#1

15000

You searched with keywords containing **(Walkab* or pedestrian* or "Land mix" or "land use mix" or traffic or road*)**
